# Investigating Variability in Metabolomics: A Comparative Study of Analytical Platforms and Blood Matrices Using HPLC-HRMS

**DOI:** 10.3390/molecules31050814

**Published:** 2026-02-28

**Authors:** Giulia Guerra, Alessio Polymeropoulos, Elisabetta Venturelli, Veronica Huber, Francesco Segrado, Daniele Morelli, Sabina Sieri

**Affiliations:** 1 Epidemiology and Prevention Unit, Fondazione IRCCS Istituto Nazionale dei Tumori di Milano, 20133 Milan, Italy; giulia.guerra@istitutotumori.mi.it (G.G.); sabina.sieri@istitutotumori.mi.it (S.S.); 2 Biostatistics for Clinical Research, Fondazione IRCCS Istituto Nazionale dei Tumori di Milano, 20133 Milan, Italy; alessio.polymeropoulos@istitutotumori.mi.it; 3 Nutrition Research and Metabolomics Unit, Fondazione IRCCS Istituto Nazionale dei Tumori di Milano, 20133 Milan, Italy; francesco.segrado@istitutotumori.mi.it; 4 Unit of Translational Immunology, Fondazione IRCCS Istituto Nazionale dei Tumori di Milano, 20133 Milan, Italy; veronica.huber@istitutotumori.mi.it; 5 Laboratory Medicine Department, Fondazione IRCCS Istituto Nazionale dei Tumori di Milano, 20133 Milan, Italy; daniele.morelli@istitutotumori.mi.it

**Keywords:** metabolomics, human plasma, blood matrix, validation, data quality

## Abstract

Untargeted metabolomics faces significant challenges in standardization due to variability introduced by sample preparation and analytical workflows. We systematically evaluated the impact of biological matrices, extraction protocols, and chromatographic configurations to establish a mechanism-informed framework aimed at improving reproducibility in large-scale clinical and epidemiological studies. Three extraction protocols were compared using an in-house pooled heparin plasma: monophasic protein precipitation with isopropanol (IPA), methanol:acetonitrile (MeOH:ACN), and a modified Matyash biphasic method. The most reproducible protocol was then applied to four blood matrices. Samples were analysed using untargeted metabolomics on hydrophilic interaction liquid chromatography (HILIC) and reversed-phase (RP) HPLC columns, with mass spectrometry data processed using Compound Discoverer. Both IPA and MeOH:ACN extractions achieved over 80% of features with coefficient of variation (CV%) ≤ 30% for both RP and HILIC, whereas the Matyash method showed higher variability, with a larger proportion of metabolites exhibiting CV% > 30%. Across matrices, RP chromatography detected over 80% of metabolites with CV% < 30%, while HILIC showed higher variability, with at least 20% of metabolites above this threshold. Among matrices, serum and heparin plasma outperformed EDTA and citrate in reproducibility. We propose a standardized workflow in which monophasic extractions combined with RP chromatography maximize reproducibility and metabolite coverage, minimizing methodological artefacts and providing a reliable framework for robust biological discovery in large-scale untargeted metabolomics studies.

## 1. Introduction

Metabolomics has emerged as a powerful analytical platform capable of detecting thousands of metabolites within biological samples. By providing comprehensive metabolic fingerprints, it enables the elucidation of pathways involved in physiological regulation, disease progression, and environmental interactions, thereby supporting both clinical research and large-scale epidemiological investigations [1,2].

Despite its potential, the field faces significant challenges in standardizing and harmonizing data across diverse laboratories and platforms, which limits reproducibility and comparability [3,4]. Untargeted metabolomics workflows, in particular, encompass multiple stages—pre-analytical, analytical, and post-analytical—each contributing to overall data variability. Pre-analytical factors, such as sample collection, anticoagulant choice, and storage, can profoundly affect metabolite stability and extraction efficiency [5].

For instance, variations in ionic strength and protein binding associated with EDTA, citrate, or heparin anticoagulant can alter metabolite recovery and ionization efficiency in mass spectrometry, introducing systematic bias that may require statistical harmonization approaches for correction [6,7].

Analytical variability further arises from differences in extraction solvents, chromatographic separation, and mass spectrometric parameters [8,9]. Solvent polarity and protein precipitation efficiency determine metabolite recovery extraction [10], while chromatographic mechanisms—such as hydrophobic interactions in reversed-phase (RP) versus polar partitioning in HILIC—modulate both coverage [11] and reproducibility [12]. Crucially, these sources of variability can interact across the workflow. Early-stage factors like matrix composition can propagate through the pipeline, being either amplified or partially mitigated by downstream processes such as chromatography or data normalization. Such cross-stage interactions are particularly critical in large-scale clinical studies and biobank-based research.

Recognizing these complexities, community-driven initiatives and consortia, such as the Metabolomics Workbench, have developed guidelines to standardize workflows and facilitate cross-study comparability [13,14,15,16,17]. Nevertheless, universally accepted procedures remain elusive due to the vast chemical diversity of the metabolome, the complexity of human biofluids, and the inherent trade-offs between metabolite coverage, reproducibility, and sensitivity. Current validation approaches often rely on reference materials, such as SRM 1950 [18,19] or yeast extracts [20], to benchmark performance. While these materials cannot capture the full complexity of every plasma pool, they offer reproducible reference points for systematic evaluation.

In this context, the present study aims to define the methodological determinants that shape the analytical window of untargeted metabolomics performed by liquid chromatography coupled with high-resolution mass spectrometry (HPLC–HRMS) in blood samples. By systematically isolating the contribution of sample extraction chemistry, chromatographic selectivity (RP versus HILIC column) and blood matrix composition, including serum and plasma collected with different anticoagulants, we sought to elucidate how pre-analytical and analytical variables influence metabolite coverage, ionization behaviour, and reproducibility. Beyond untargeted profiling, a targeted assessment using a reference metabolite panel was incorporated as a qualitative benchmark. This approach, applied to in-house pooled plasma samples and supported by the analysis of a certified yeast reference material, enabled an orthogonal evaluation of analytical consistency and workflow robustness.

Through this integrated strategy, this study aims to provide a mechanistically informed framework for workflow optimization and harmonization in large-scale clinical and epidemiological metabolomics.

## 2. Results

Variability arising from key steps in the metabolomics workflow was assessed, including sample extraction, chromatographic conditions, and matrix selection. To evaluate differences in metabolomic profiles across blood matrices, serum, plasma heparin, plasma EDTA, and plasma citrate were analysed, each in triplicate. Analytical coverage was further extended by employing two complementary chromatographic platforms: reversed-phase (RP) and hydrophilic interaction liquid chromatography (HILIC) columns, both acquired in positive ionization mode.

### 2.1. Sample Extraction Protocols

#### 2.1.1. Comparison of Extraction Protocols for Plasma Metabolomics Using Both RP and HILIC Chromatography Platforms

For comparison and validation of the extraction step of the metabolomics platform, an in-house pooled plasma heparin was prepared, and three different liquid–liquid extraction (LLE) protocols were selected for the sample preparation step. Unsupervised multivariate analyses were performed to evaluate the impact of the three different sample extraction protocols on the global metabolomic profiles. Principal component analysis (PCA) and two-way hierarchical clustering were applied to data acquired in positive ionization mode using both RP and HILIC chromatography columns.

**Figure 1:**
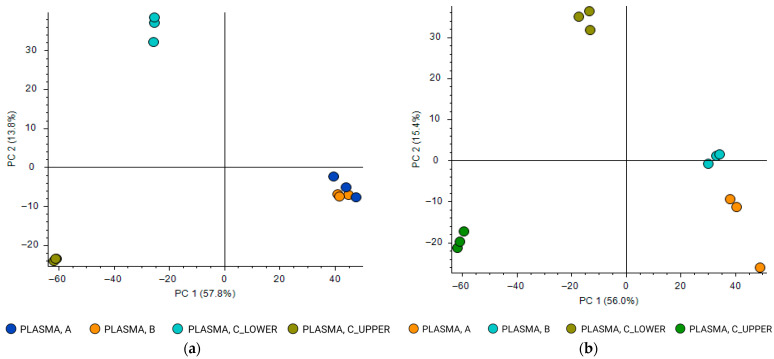
Principal component analysis (PCA) of the metabolomics dataset obtained from a pooled plasma heparin sample prepared using different extraction protocols and processed with Compound Discoverer. Four different extracts were compared: protocol A, LLE extraction by IPA; protocol B, LLE extraction by MeOH/ACN mixture; protocol C, modified Matyash extraction (two phases: polar lower phase and MTBE-nonpolar upper phase). Data were acquired in positive ionization mode: (**a**) HPLC analysis on a reversed-phase (RP) column; (**b**) HPLC analysis on a hydrophilic interaction liquid chromatography (HILIC) column.

Principal component analysis (PCA) performed on RP data (Figure 1a) showed that samples extracted using protocol A (IPA-based LLE) and protocol B (MeOH/ACN-based LLE) clustered closely together, indicating highly comparable metabolomic profiles. In contrast, both phases obtained from protocol C (polar lower phase and MTBE-nonpolar upper phase) [21] were clearly separated from protocols A and B along the principal components, suggesting that this protocol introduced a distinct metabolic signature under RP conditions.

When HILIC separation was applied (Figure 1b), PCA revealed a clear separation among all three extraction protocols. Protocols A, B, and C formed well-defined and distinct clusters, indicating that HILIC chromatography enhanced the discrimination of metabolomic profiles resulting from these different sample preparation strategies.

**Figure 2:**
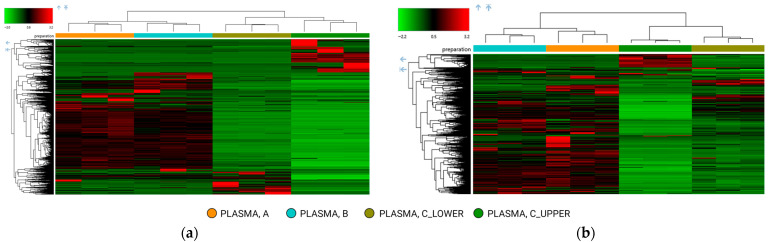
Two-way hierarchical clustering analysis using Euclidean distance of metabolomics dataset. The heatmap shows the four different extracts obtained from a pooled plasma heparin: protocol A, LLE extraction by IPA; protocol B, LLE extraction by MeOH/ACN mixture; protocol C, modified Matyash extraction (two extracts: polar lower phase and MTBE-nonpolar upper phase). Data were acquired in positive ionization mode: (**a**) HPLC analysis on a reversed-phase (RP) column; (**b**) HPLC analysis on a hydrophilic interaction liquid chromatography (HILIC) column.

These observations were further supported by two-way hierarchical clustering analysis (Figure 2). Under RP conditions (Figure 2a), protocols A and B were not segregated into distinct clusters, confirming the similarity of their metabolomic patterns across the detected features, whereas the polar and nonpolar fractions obtained with protocol C clustered separately. A similar clustering pattern was observed under HILIC conditions (Figure 2b). Protocols A and B again showed limited separation, whereas the two fractions derived from protocol C were clearly distinguished, reflecting reproducible differences in metabolite composition.

To further investigate the overlap and specificity of metabolite detection, Venn diagram analyses were performed considering only features consistently detected across all three replicates for each protocol and putatively identified by Compound Discoverer (Figure S4).

In RP mode (Figure S4a), protocols A and B shared 678 putatively annotated metabolites, indicating nearly identical metabolite coverage. In contrast, comparisons between protocols A or B and the polar phase of protocol C revealed fewer than 250 shared metabolites. As expected, the overlap between the polar and nonpolar phases of protocol C was minimal (Figure S4c,d), highlighting a strong partitioning of metabolites between the two fractions.

A similar trend was observed in HILIC analyses (Figure S4b), where protocols A and B shared 328 metabolites, while substantially fewer metabolites were shared between these protocols and the polar fraction of protocol C. These results indicate that monophasic extraction protocols provide more consistent metabolite coverage across chromatographic platforms, whereas biphasic extraction leads to a pronounced redistribution of metabolites.

#### 2.1.2. Analytical Reproducibility of Extraction Protocols

The analytical robustness of each extraction protocol was evaluated using the coefficient of variation (CV%) of peak areas across triplicates, considering all detected features (Figure S5). Protocols A and B demonstrated the highest reproducibility, with more than 80% of features exhibiting CV% ≤ 30% in both RP and HILIC modes.

In contrast, protocol C showed reduced repeatability, particularly for the nonpolar upper fraction, which exhibited a markedly higher proportion of features exceeding the accepted variability threshold. These results indicate that monophasic extraction strategies offer superior analytical precision under the tested conditions.

#### 2.1.3. Targeted Reference Panel: Evaluation of Extraction Performance

To validate the findings from the untargeted analyses, a targeted evaluation was performed using the SRM 1950 NIST-derived benchmark panel of 75 consensus metabolites on the home-made plasma samples. The certified yeast reference material benchmark panel of 91 metabolites was applied on the yeast (results are provided in the Supplementary File). This evaluation focused on detection rates, analytical reproducibility, and chromatographic selectivity across different extraction protocols and chromatographic modes (RP and HILIC).

**Figure 3:**
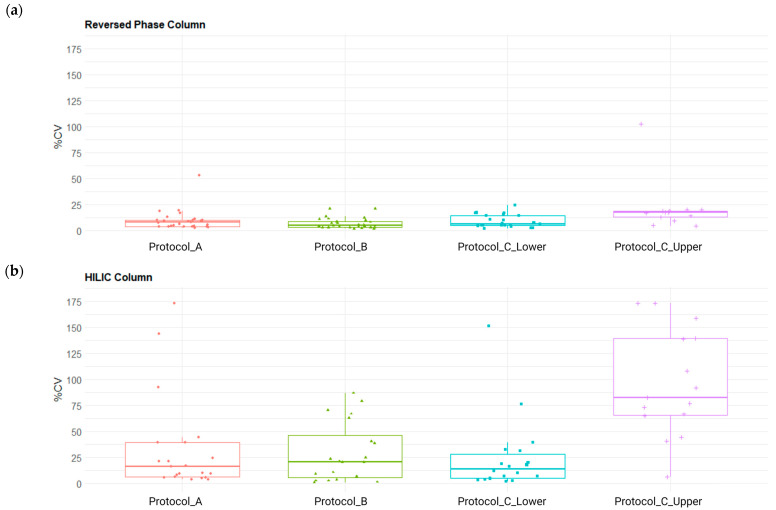
Box plot comparison of the coefficient of variation (CV%) distributions for compounds included in the SRM 1950 panel across different pooled plasma heparin extraction protocols: protocol A, LLE extraction with isopropanol (IPA); protocol B, LLE extraction with a methanol/acetonitrile (MeOH/ACN) mixture; and protocol C, modified Matyash extraction (two phases: polar lower phase and MTBE-nonpolar upper phase). Data were acquired in positive ionization mode: (**a**) HPLC analysis on a reversed-phase (RP) column; (**b**) HPLC analysis on a hydrophilic interaction liquid chromatography (HILIC) column.

In RP mode, protocols A and B achieved the highest detection rates, identifying 27/75 and 28/75 compounds, respectively, with low median CV% values of 7.9% and 4.8% (Figure 3). Although detection rates were slightly lower under HILIC conditions, protocols A and B maintained acceptable reproducibility. In contrast, protocol C_upper exhibited a markedly elevated median CV% of 82.2%, indicating poor reproducibility under these conditions.

### 2.2. Blood Matrix Comparison

#### 2.2.1. Comparison of Blood Matrix Metabolomics Using IPA Protocol and Both RP and HILIC Chromatography Platforms

**Figure 4:**
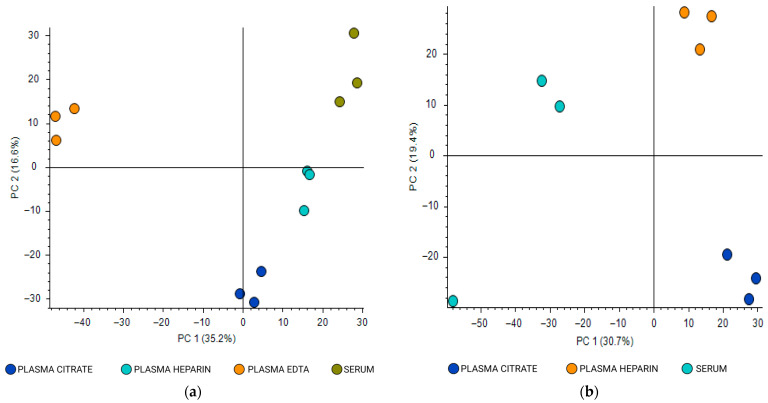
Principal component analysis of metabolites shared among four different blood matrices of the same donors (pooled samples): plasma heparin, plasma citrate, plasma EDTA, and serum. Data were acquired in positive ionization mode: (**a**) HPLC analysis on a reversed-phase (RP) column; (**b**) HPLC analysis on a hydrophilic interaction liquid chromatography (HILIC) column.

Unsupervised multivariate analyses of untargeted metabolomics revealed distinct metabolic profiles among the four blood matrices (plasma heparin, plasma citrate, plasma EDTA, and serum). Principal component analysis (PCA) demonstrated clear separation and tight clustering of metabolites according to matrix (Figure 4).

**Figure 5:**
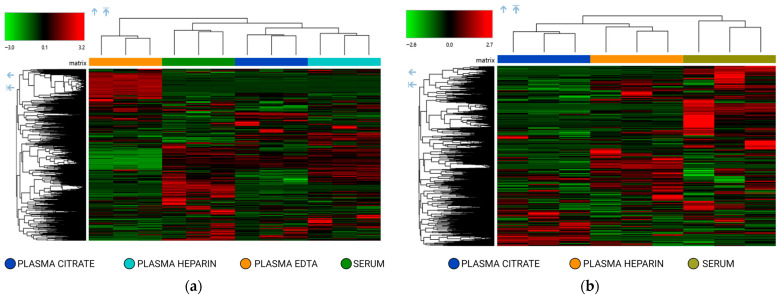
Two-way hierarchical clustering analysis using Euclidean distance of metabolomics data. The heatmap shows the clustering of the metabolites detected in pooled plasma constituted by the same donors of four different matrices: plasma heparin, plasma citrate, plasma EDTA, and serum. Data were acquired in positive ionization mode: (**a**) HPLC analysis on a reversed-phase (RP) column; (**b**) HPLC analysis on a hydrophilic interaction liquid chromatography (HILIC) column.

Hierarchical clustering further confirmed systematic variations between groups (Figure 5). In RP mode (Figure 5a), metabolites from plasma heparin, plasma citrate, plasma EDTA, and serum formed clearly separated clusters, indicating that less polar metabolites are differentially captured depending on the matrix.

The HILIC analysis revealed clustering patterns that highlight the selective detection of highly polar metabolites. Indeed, serum, heparin, and citrate displayed divergent metabolic fingerprints (Figure 5b). It should be noted that the plasma EDTA matrix was not investigated using the HILIC setting due to significant signal interference caused by the EDTA additive.

These findings confirm that the matrix-driven variability impact untargeted metabolomics analysis.

To evaluate the overlap of the metabolome across matrices, a Venn diagram (Figure S6) was employed to compare putatively identified analytes detected in 100% of triplicate. In the RP acquisition mode, 386 metabolites were commonly detected across serum, plasma citrate, and plasma heparin. This shared subset represents over 75% of all uniquely annotated metabolites identified across these three matrices, suggesting a high degree of core metabolite consistency despite the matrix-specific variations observed in the multivariate models. Similar comparative trends were observed with the HILIC column, highlighting the influence of both the chromatographic setting and the anticoagulant on the final metabolic coverage. When using the HILIC column (Figure S6a), the overlap between matrices was markedly reduced: only 73 metabolites were shared across all four matrices, representing less than 20% of the annotated metabolites identified in each biofluid. Despite this lower overall concordance, serum and plasma heparin exhibited a high degree of similarity, sharing 305 metabolites (corresponding to ~80% and ~75% of their identified features, respectively). Plasma citrate showed the largest number of putatively identified metabolites in HILIC mode (325), accounting for ~67% of all annotated metabolites detected in this matrix.

#### 2.2.2. Analytical Reproducibility of Blood Matrix Samples

The analytical robustness of each blood matrix sample was evaluated using the coefficient of variation (CV%) of peak areas across triplicates, considering all detected features (Figure S7). The CV% of the matrices analysed by RP is less than 30% for more of the 80% of the metabolites detected in each matrix. HILIC analysis showed a higher variability because at least the 20% of the features have a CV% > 30 (Figure S7 and Table S3).

#### 2.2.3. Targeted Reference Panel: Evaluation of Blood Matrix Performance

**Figure 6:**
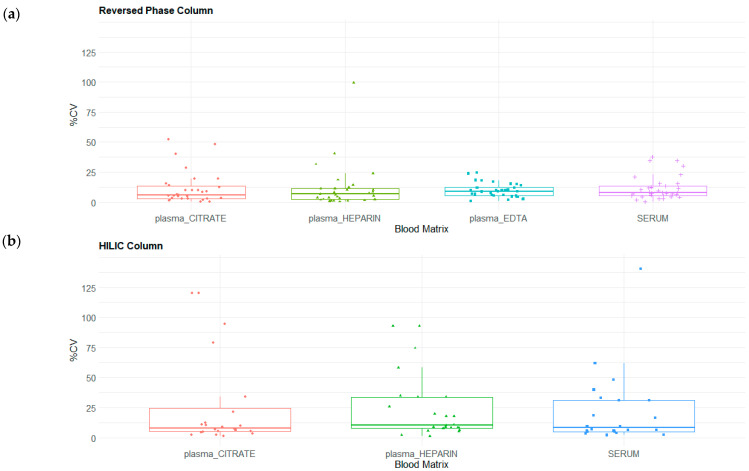
Box plot comparisons of coefficient of variation (CV%) distribution for compounds included in the SRM panel and detected in triplicates for each matrix (plasma heparin, plasma citrate, plasma EDTA, and serum). Data were acquired in positive ionization mode: (**a**) HPLC analysis on a reversed-phase (RP) column; (**b**) HPLC analysis on a hydrophilic interaction liquid chromatography (HILIC) column.

The evaluation of the 75 reference analytes across the different blood matrices demonstrated high consistency in metabolite detection and analytical precision. In RP mode, the detection rates remained remarkably stable across matrices, with 33 metabolites identified in plasma citrate, plasma EDTA, and serum and 32 in heparinized plasma. These detections were accompanied by robust median CV% values of 6.4%, 9.3%, 6.9%, and 7.4%, respectively (Figure 6). Although detection rates were slightly lower in HILIC mode, consistent results were maintained with 24 features detected in both citrate and heparinized plasma and 22 in serum. The corresponding median CV% values in HILIC mode were 9.5%, 10.4%, and 8.2%, respectively (Figure 6). Collectively, these data indicate that serum and heparinized plasma provide the highest analytical stability, exhibiting the lowest overall signal variability across both chromatographic acquisition modes.

To assess how anticoagulants influence compounds ionization, six different adducts were monitored for each molecule. For every compound, the following adducts were examined in positive mode: [M]^+^, [M+H]^+^, [M+K]^+^, [M+Na]^+^, [M–H_2_O+H]^+^, and [M+NH_4_]^+^, whereas [M+Cl]^−^ adduct was evaluated in negative mode only for glucose compound. In Table 1, only the predominant adducts and the relevant matrix-dependent differences are reported. Detailed adduct distributions are provided in the Supplementary_file (Supplementary Adduct_Hilic file).

The analysis performed with RP column shows [M+H]^+^ as the most abundant adduct, except for most of the amino acids detected (tyrosine, phenylalanine, valine), creatinine, bilirubin, retinol, 25-Hydroxyvitamin D3, and cholesterol, with high percentages of [M+Na]^+^ and [M]^+^ adduct, while for methionine, zeaxantin, 4-pyridoxic, progesterone, and lutein, [M+NH_4_]^+^ was the most abundant adduct. All the adducts have similar percentages of formation for the 4 different matrices, except for methionine, where EDTA has higher formation of [M+H]^+^; arginine, where in plasma EDTA no [M+K]^+^ formation was observed; and lutein, where a different adduct composition was observed for serum samples and no detection for the plasma heparin sample. Creatinine and valine have a 27.7 and 19.4% of [M+K]^+^ adduct, respectively, in contrast with 4–7% for the other anticoagulant.

The analysis performed by the HILIC column showed the [M+H]^+^ ion was the most abundant adduct for most compounds, with the exception of glucose, which showed higher signal in negative mode with the formation of the [M+Cl]^−^ adduct. For tocopherol, lutein, zeaxanthin, 4-pyridoxic acid, and fatty acids, the most abundant species were [M]^+^ and [M+NH_4_]^+^. Overall, adduct formation percentages were similar across the four matrices, except for lutein and zeaxanthin, which had 100% [M+Na]^+^ formation in serum compared with 0% in the others. Testosterone had increased [M+Na]^+^ formation in plasma citrate, with an 18% contribution, whereas only 12% and 4% were observed in heparinized plasma and serum, respectively.

### 2.3. Targeted Panel Profiling Using Plasma Heparin, IPA Protocol, and Both RP and HILIC Chromatography Platforms

**Figure 7:**
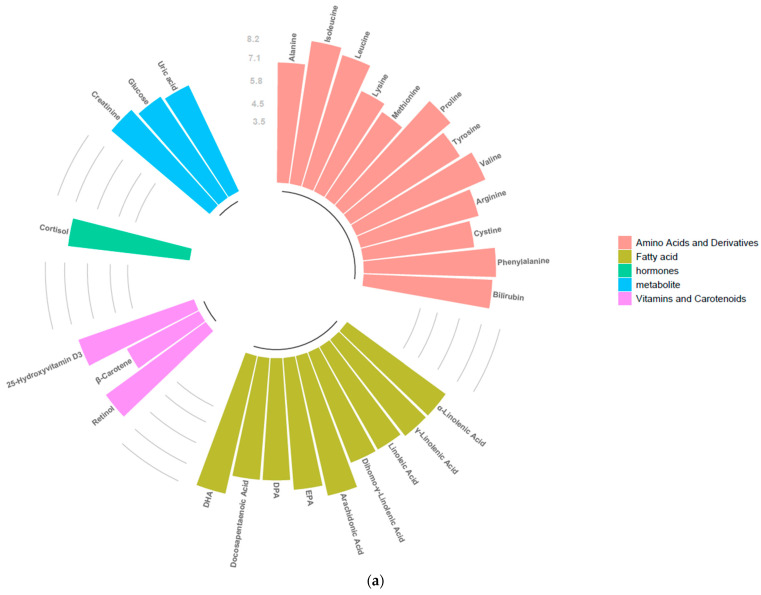

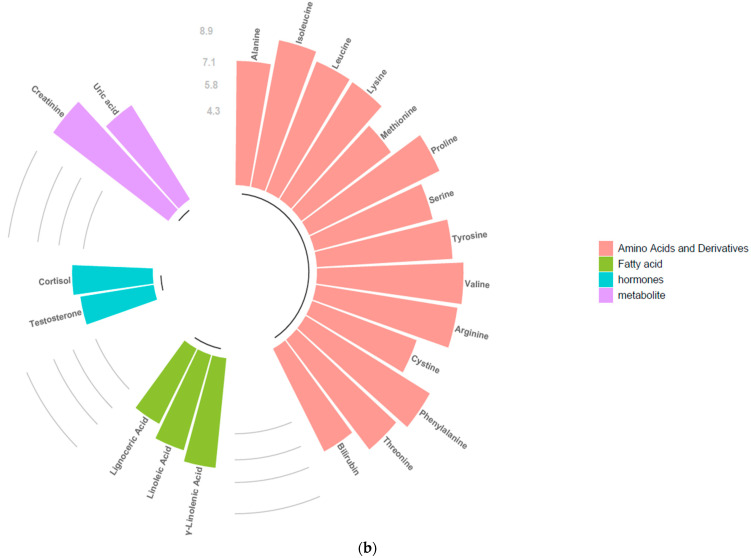
Circular bar plot showing molecules detected in plasma heparin using protocol A (IPA), expressed as log_10_ median peak area. Data were acquired in positive ionization mode: (**a**) HPLC analysis on a reversed-phase (RP) column; (**b**) HPLC analysis on a hydrophilic interaction liquid chromatography (HILIC) column.

The molecular coverage in plasma heparin is illustrated in Figure 7, where detected analytes are categorized into five primary chemical classes according to SRM 1950 classification: amino acids and derivatives, fatty acids, hormones, metabolites, and vitamins and carotenoids.

The RP chromatographic platform enabled the detection of a broad range of hydrophobic and amphiphilic compounds, as shown in Figure 7a. Within the fatty acid class, extensive coverage was observed for long-chain polyunsaturated fatty acids, including docosahexaenoic acid (DHA), docosapentaenoic acid (DPA), eicosapentaenoic acid (EPA), arachidonic acid, and linoleic and α-linolenic acids. This mode was also effective in capturing fat-soluble vitamins and carotenoids, specifically retinol, β-carotene, and 25-hydroxyvitamin D3. Regarding amino acids and derivatives, eleven species were identified, with isoleucine and alanine exhibiting the highest log_10_ median peak areas, exceeding 7.1 log_10_. Furthermore, cortisol, glucose, uric acid, and creatinine were successfully detected within this analysis.

In contrast, the HILIC platform provided a distinct metabolic profile focused on polar analytes, as depicted in Figure 7b. A total of 13 amino acids and derivatives were identified. Notably, serine and threonine were detected in this mode, while isoleucine, alanine, and leucine maintained high signal intensities. The hormonal profile was expanded in HILIC mode to include both cortisol and testosterone, the latter being uniquely identified by this stationary phase. For the polar metabolite fraction, uric acid and creatinine demonstrated high signal intensities, with log_10_ median peak areas approaching 8.9 log_10_. However, fatty acid coverage was significantly reduced compared to the RP analysis, with only lignoceric, linoleic, and γ-linolenic acids reaching the detection threshold.

## 3. Discussion

This study systematically evaluated the sources of variability introduced at key stages of untargeted metabolomics workflows, including, sample extraction, chromatographic settings, and matrix selection. Our findings underscore that the architecture of an untargeted metabolomics workflow is not merely a technical choice, but a primary determinant of the biological narrative captured. By systematically evaluating the interaction between extraction, chromatography, and matrix selection, we showed how analytical variables define the boundaries of the detectable metabolome.

Metabolite extraction emerged as one of the most influential determinants of analytical performance. The comparison between monophasic protocols (IPA and MeOH/ACN) and the biphasic modified Matyash extraction [21] clearly showed that solvent architecture governs both metabolite coverage and reproducibility. Monophasic extractions generated highly overlapping metabolic profiles and tight clustering in multivariate models across both RP and HILIC platforms, compared to the biphasic Matyash-modified method. The tight clustering of protocols A and B in the PCA models suggests that these solvent systems, despite differences in polarity and protic character, exert a similar effect on the blood matrix. These solvents effectively precipitate proteins while maintaining a wide range of extracted metabolites. Conversely, protocol C introduced a distinct metabolic signature, likely due to the physical partitioning of metabolites between the two phases. Matyash protocol was usually proposed as an alternative to monophasic extraction [22] for untargeted metabolomics and lipidomics applications [23,24], since it may enhance mass spectrometric detection of certain compound classes by removing the nonpolar fraction. However, our findings highlight important limitations. While biphasic systems are theoretically advantageous for separating lipids from aqueous metabolites, our data suggest that this partitioning compromise reproducibility. The clear separation of protocol C in multivariate analyses, together with its higher variability (CV% > 30%), indicates that phase separation may introduce stochastic effects in analyte recovery [10]. These observations are consistent with previous reports [25]: Martias et al. [8] described a median CV% of 17% for MeOH/ACN/H_2_O extractions, Ang et al. [26] observed CV% of 3–11% for select metabolite classes, and Sitnikov et al. found reduced reproducibility for MTBE-based protocols, likely attributable to the irreproducible partitioning of certain metabolites [10]. Together with our results, this literature supports monophasic extraction as the most robust strategy for discovery-driven metabolomics, particularly in large-scale clinical cohorts where subtle biological differences must be distinguished from technical noise [27].

Beyond extraction, chromatographic separation strongly influenced metabolite detection patterns. The complementarity between RP and HILIC chromatography highlights a fundamental trade-off in metabolomic depth [28].

RP chromatography remains the cornerstone of robustness, offering tight peak areas and consistent detection of mid-to-nonpolar species [29,30]. Peak area stability was consistently higher under RP conditions, reflecting its well-established robustness and reduced sensitivity to minor fluctuations in mobile phase composition.

HILIC, in contrast, expanded the analytical window toward highly polar metabolites, However, this increased polar coverage came with slightly higher variability. Retention in HILIC depends on partitioning into a water-rich stationary phase layer, making it more sensitive to matrix composition, salt concentration, and solvent fluctuations [31,32]. Early elution and limited retention of highly polar compounds in RP, and poor retention of hydrophobic analytes in HILIC, further illustrate the complementary but selective nature of the two platforms.

Blood matrix selection significantly shaped metabolic fingerprints [7]. Distinct clustering of serum, plasma heparin, plasma EDTA, and plasma citrate demonstrated that the “chemical background” of the samples influences both metabolite detection and ionization efficiency, which may ultimately bias biological conclusions.

Our data showed that the evaluated matrices were generally suitable for metabolomics analyses. However, the distinct metabolic signatures of EDTA, citrate, and heparin plasma are not just reflections of endogenous biology, but largely due to products of ion suppression and altered ionization efficiency.

EDTA caused faster degradation of the HPLC column compared with the other matrices [33] and exhibited a pronounced carryover effect in blank samples, consistent with the findings of Pereira et al. [34], who reported a large residual chromatographic peak after 30 consecutive plasma EDTA injections. The use of EDTA in HPLC–HRMS analysis remains controversial. Some studies prefer EDTA over heparin anticoagulant because the first can introduce chemical noise in mass spectra [35], whereas others report that EDTA-induced ion suppression makes heparin a more reliable choice [36].

In addition, the significantly higher ionic strength of sodium citrate and potassium EDTA can alter ionization efficiency, leading to metabolite-specific ion suppression or enhancement compared with plasma heparin and serum. These matrix effects result from competition between analytes and co-eluting species during ion formation, where differences in ionization energy and proton affinity determine which ions are preferentially generated. Consistent with our study, Gonzalez et al. [37] reported that all phospholipid species’ peak areas are highest in plasma EDTA. They suggested that the difference in ionic strength between the anticoagulant can affect the extraction recovery. Indeed, the ionic strengths of sodium citrate, potassium EDTA, and lithium heparin are 0.209, 0.090, and 0.000014 mol/dm^3^, respectively. Higher ionic strength can enhance protein denaturation and promote lipid release from lipoproteins. Moreover, in our experience, high levels of sodium can cause clog in the instrument source, leading to more frequent cleaning procedure, and so more frequent machine downtime. To overcome this problem in large-scale studies, a higher dilution factor of plasma citrate sample will be applied. Our findings agree with Barri et al. [33] who recommend plasma heparin and serum as the suitable matrices for HPLC-MS metabolomics analysis.

Furthermore, the impact of various biological matrices and anticoagulants on adduct formation was assessed using RP and HILIC platforms. Indeed, the varying salt compositions across these matrices can affect adduct formation. Analyte ionization, mobile phase salts, and instrument settings all contribute to the types of adducts formed in the ESI source [38]. Overall, [M+H]^+^ was the predominant adduct for most compounds, while selected amino acids, creatinine, lipophilic vitamins, and steroids showed higher contributions of [M+Na]^+^, [M+K]^+^, or [M+NH_4_]^+^ depending on the compound’s chemical properties. Matrix effects were generally limited, but notable exceptions included methionine, arginine, lutein, creatinine, valine, and testosterone, which displayed altered adduct patterns or were undetectable in specific matrices. While no major shifts were observed among the different anticoagulants, plasma from citrate and EDTA showed a predictable increase in [M+Na]^+^ and [M+K]^+^ adducts, respectively, consistent with their sodium and potassium content. We observed this phenomenon particularly in RP chromatography for amino acids, likely because their high hydrophilicity results in co-elution with salts, thereby promoting alternative adduct formation over protonated species.

HILIC chromatography largely confirmed these trends, with glucose showing preferential formation of [M+Cl]^−^ in negative mode. According to the literature, these findings highlight that while overall adduct formation is robust across matrices, compound-specific and matrix-dependent ionization differences exist [33]. Awareness of these patterns is essential for accurate quantification, method optimization, and reproducibility in targeted and untargeted metabolomics, particularly when analysing biobanked samples.

Altogether, the differences observed among extraction protocols, chromatographic systems, and biological matrices can be therefore explained by chemical and biological mechanisms. Monophasic extraction methods, such as IPA and MeOH/ACN, promote rapid and uniform protein precipitation [39,40], minimizing metabolite loss and improving reproducibility. Meanwhile, biphasic protocols rely on physical separation of polar and nonpolar phases, which is sensitive to subtle variations in sample composition and handling, resulting in lower metabolite overlap and higher variability. Chromatographic behaviour further modulates these effects: reversed-phase columns favour retention of nonpolar and moderately polar metabolites, yielding broader coverage and more consistent measurements, while HILIC columns depend on partitioning into a water-rich layer, making retention more sensitive to solvent composition, salts, and matrix effects. Matrix composition, particularly anticoagulant type, influences ionic strength and salt composition [41], protein binding, lipid release, and electrospray ionization efficiency, with high sodium or potassium levels in plasma EDTA and plasma citrate enhancing protein denaturation and lipid extraction but also increasing ion suppression and modifying adduct formation [37]. Collectively, these mechanistic factors account for the observed differences in metabolite coverage, reproducibility, and analytical robustness, providing a chemical and biological rationale for the systematic patterns identified in our methodological comparisons.

Despite the insights gained from this systematic evaluation, some limitations must be acknowledged.

A primary constraint was the absence of a comprehensive library of authentic standards for every detected feature. While we utilized the NIST SRM 1950 metabolite list as a benchmark to facilitate annotation and ensure comparability with the literature, the reference material itself was not analysed directly, and level-one annotation was not achieved for the analytes. This lack of metabolite-specific standards in our in-house plasma pools precluded a rigorous quantitative assessment of absolute recovery and the determination of lower limits of quantification (LLOQ). However, this was partially mitigated by prioritizing annotations and utilizing a commercially available yeast reference material to provide a robust assessment of analytical reproducibility (CV%), as detailed in the Supplementary Information. Ultimately, while these factors limit absolute quantification, they do not compromise this study’s primary objective: providing a systematic mechanistic comparison of extraction methods, chromatographic conditions, and biological matrices.

Furthermore, this study focused predominantly on positive ionization mode. While RP and HILIC platforms provided broad coverage, extending the investigation to negative ionization would be necessary to fully characterize certain metabolite classes, such as organic acids and specific lipid species, which may exhibit different matrix-dependent ionization behaviours. Additionally, while the impact of anticoagulants on adduct formation was documented, the underlying mechanism of ion suppression remains a complex interplay between co-eluting matrix components and the ESI source dynamics, which may vary across different instrument manufacturers and ion source designs.

Finally, while our findings highlight the robustness of monophasic extractions, it is important to note that specialized applications—such as targeted lipidomic or the analysis of extremely hydrophobic signalling molecules—might still benefit from the partitioning offered by biphasic methods, provided that the increased variability is accounted for in the study design. These limitations underscore that while a standardized workflow using monophasic IPA or MeOH/ACN and RP chromatography is optimal for general discovery-driven metabolomics, researchers must remain mindful of the inherent selectivity of the analytical window and the specific chemical requirements of their target analytes.

In conclusion, this study systematically evaluated key sources of variability in untargeted metabolomics workflows. Monophasic extractions with IPA or MeOH/ACN emerged as the most robust strategies, particularly suitable for large-scale studies due to their superior reproducibility compared to biphasic methods. While RP chromatography offered higher peak stability and robustness, HILIC remains essential to capture the polar metabolome, despite its higher sensitivity to matrix effects. Furthermore, our findings indicate that while all matrices are viable, serum and heparin plasma are preferable to minimize salt-related adduct interference and instrument downtime. Overall, our results establish a mechanism-informed framework supported by reference materials (NIST benchmark and yeast controls), showing that combining monophasic extractions with RP chromatography achieves an optimal balance between metabolite coverage and analytical stability. This framework offers a standardized roadmap to minimize methodological artefacts, supporting reliable and reproducible biological discovery in large-scale clinical and epidemiological studies.

## 4. Materials and Methods

### 4.1. Reagents and Materials

Ultrapure water (H_2_O, purity of 18.2 MΩ cm^−1^) was obtained by the PURELAB Ultra Purification System (ELGA LabWater, Lane End, High Wycombe, UK). Liquid chromatography mass spectrometry (HPLC-HRMS) grade 2-propanol (IPA), acetonitrile (ACN), HPLC-HRMS grade formic acid (FA), HPLC-HRMS grade methanol (MeOH), and methyl tert-butyl ether (MTBE) were supplied by Carlo Erba (Carlo Erba Reagents S.r.l., Milan, Italy). Ammonium acetate was provided by Sigma (Milan, Italy). Phosphocholine-d9 chloride calcium salt (tetrahydrate) was purchased from Spectra 2000 s.r.l. (Rome, Italy). Metabolite yeast extract (unlabelled) ISO1-UNL lot. N. 20220615 2022.06.15 was purchased from Cambridge Isotope Laboratories (Andover, MA, USA).

### 4.2. Solution Preparation

The internal standard (I.S.) consisted of 20 mL of choline-d9 with a concentration of 100 ng/mL in H_2_O:MeOH (1:1, *v*/*v*), and stored at −20 °C. The isopropyl alcohol (IPA), which is the protein precipitation solvent for protocol A, was stored at −20 °C in a freezer until analysis. The extraction solvent of protocol B is constituted by ACN:MeOH (1:1, *v*/*v*) and stored at room temperature.

### 4.3. Blood Sample Collection

For comparison and validation tests of the metabolomics platform, in-house pooled plasma was constituted from leftover heparinised plasma samples obtained from women who provided consent for its use [42]. Samples were collected after 3 h at room temperature and were stored at −80 °C for 5 years. As only anonymized patient samples were used, which were not obtained through investigator intervention or interaction with the individuals, the investigators cannot ascertain the identity of the subjects.

To compare matrix effects on metabolomics analysis, samples from four different blood matrices were collected from three healthy volunteers. All subjects gave their informed consent to use their blood sample for research purpose. For each subject, plasma EDTA, plasma citrate, plasma heparin, and serum were collected from the same venepuncture. Samples were collected after 3 h at room temperature. To obtain comparable pools of the different matrices, 200 µL of each matrix from each subject was used. All pooled samples were aliquoted and stored at −80 °C until analysis.

### 4.4. Sample Extraction

#### 4.4.1. Liquid–Liquid Extraction with IPA (Protocol A)

Protocol A is a modified version of Segrado et al. [43]’s sample preparation. In brief, an aliquot of 200 µL of pooled plasma was added with 10 μL of Choline-d9 I.S. and 600 µL of cold protein precipitation solvent, then the mixture was vortexed for 30 s. The protein precipitation was achieved by centrifugation at 13,300 rpm at 4 °C for 15 min. After that, the supernatant was evaporated at vacuum centrifuge at 35 °C overnight. Next, 100 µL of IPA:H_2_O (1:1, *v*/*v*) was added, vortexed, and finally dispensed in a glass autosampler vial for subsequent analysis.

#### 4.4.2. Liquid–Liquid Extraction with MeOH/ACN (Protocol B)

Protocol B follows the method described by Flasch et al. [44]’s sample preparation. In brief, an aliquot of 200 μL of in-house pooled heparinized plasma was mixed with 10 μL Choline-D9 I.S. and vortexed for 30 s. Then, the samples were mixed with 760 μL of MeOH:ACN (1:1 *v*/*v*) extraction solvent and vigorously vortexed for 30 s. The samples were sonicated on bath-ice for 10 min and stored at −20 °C for 2 h. Then, the cold samples were centrifuged at 13,300 rpm at 4 °C for 15 min, and 960 μL of the supernatant was transferred to a new tube. A preconcentration was obtained by solvent evaporation in a vacuum centrifuge at 35 °C overnight. Next, 100 μL IPA:H_2_O (1:1, *v*/*v*) was added, vortexed, and centrifuged at 4 °C for 10 min. Finally, the supernatants were transferred to HPLC vials for subsequent analysis.

#### 4.4.3. Modified Matyash Extraction (Protocol C)

The modified Matyash protocol [21] was designed to obtain a biphasic mixture where both phases are analysed. The extraction was obtained by adding 2.66 mL of MTBE to 200 µL of plasma with 10 μL Choline-D9 I.S and mixing the mixture in the shaker (Fisher) (800 rpm, 10 min). Phase separation was then induced by adding 0.4 mL of water. Samples were then mixed again in the shaker (800 rpm, 5 min), incubated at 18 °C for 10 min to allow phase separation, and centrifuged at 13,300 rpm at 4 °C for 15 min. An aliquot of 300 µL of the lower polar fraction (lower phase) and 1500 µL of the upper nonpolar fraction (upper phase) were aliquoted into Eppendorf tubes and dried in a vacuum centrifuge at 35 °C overnight. The residues were reconstituted in 100 μL IPA:H_2_O (1:1, *v*/*v*), vortexed, and centrifuged at 4 °C for 10 min. Finally, the supernatants were transferred to HPLC vials for subsequent analysis.

### 4.5. HPLC-HRMS Setup

#### 4.5.1. Reversed-Phase (RP) Setup

HPLC-HRMS analyses were performed using the Ultimate 3000 RSLC (Thermo Fisher Scientific, Waltham, MA, USA) HPLC. The RP column used was Atlantis™ Premier BEH C18 AX 2.5 µm, 100 × 2.1 mm (Waters Corporation, Milford, MA, USA) with a guard-column with equal stationary phase (2.5 µm, 5 × 2.1 mm). The column temperature was set to 45 °C and a constant flow rate of 350 µL min^−1^ was applied for the whole duration of the analysis. The aqueous phase was constituted by 0.1% *v*/*v* formic acid in bidistilled water (phase B). The organic phase was 100% acetonitrile (phase C). The solvent gradient was reported in Table 2, and 2 µL was the injection volume of the samples. The total run time for analysis was 48 min.

#### 4.5.2. Hydrophilic Interaction Chromatography (HILIC) Setup

For the analysis of polar metabolites, an Atlantis Premier BEH Z-HILIC VanGuard FIT Column (2.5 µm, 2.1 mm × 100 mm) (Waters Corporation, Milford, MA, USA) was selected. The column oven temperature was maintained at 40 °C. Chromatographic separation was achieved using a binary mobile phase system composed of 10 mM acetate buffer (pH 6.6, Phase A) and 100% acetonitrile (Phase C). A 25 min multi-step gradient was implemented as detailed in Table 3, maintaining a constant flow rate of 350 µL/min. For all analyses, an injection volume of 2 µL was employed. The total run time for analysis was 25 min.

Samples were analysed in positive acquisition by reversed-phase liquid chromatography, referred to as RP, and analyses performed by HILIC chromatography are referred as HILIC.

#### 4.5.3. Instrument Setup for HRMS Analysis in Positive Mode

The HPLC system was coupled to a Thermo Scientific LTQ Orbitrap Elite mass spectrometer equipped with a Heated Electrospray Ionization (H-ESI) source.

ESI spray voltage was set to 3.5 kV. The vaporizer temperature was set to 320 °C, while the ion transfer tube was set at 300 °C. Sheath, aux, and sweep gases were set to 40, 10, and 0 arbitrary units, respectively. The full width half maximum (FWHM) resolution for the MS1 mode was 120,000 at *m*/*z* 400 in the 80–1100 *m*/*z* range. The 60,000 FWHM resolution was applied to the MS2. The top 5 *m*/*z* signals were fragmented in Higher-energy Collisional Dissociation (HCD), with a Normalized Collision Energy (NCE) values of 40 and 60% before and after RT = 25 min, respectively. The ion injection time for the MS1 mode was 50 ms, and the Automatic Gain Control (AGC) target was set to 106. MS2 ion injection time was 50 ms, while AGC target value was 105. Data were acquired with Xcalibur 2.2 (Thermo Fisher Scientific).

#### 4.5.4. Analytical Procedure

Prior to each analysis, three blank samples were run. To avoid fragmentation of background ions, the 100 most intense peaks detected in the blanks were added to the exclusion list for the Data Dependent Analysis (DDA) method. Blank samples were run every ten plasma samples to keep the chromatographic system clean and to monitor and prevent carry-over effects.

#### 4.5.5. Data Processing and Feature Extraction

Raw LC–MS data files acquired using Xcalibur 2.2 (Thermo Fisher Scientific) were processed with Compound Discoverer version 3.3 (Thermo Fisher Scientific), a software platform specifically designed for untargeted metabolomics data processing. The processing workflow, summarized in Figure S3 (Supplementary Information), included chromatographic time-range selection, peak detection, retention time alignment, and feature grouping.

Chromatographic intervals were defined according to the separation mode and set to 0.2–43 min for reversed-phase (RP) analyses and 0.2–23 min for hydrophilic interaction liquid chromatography (HILIC) analyses. Feature detection was performed using the Detect Compounds node, with a minimum peak intensity threshold of 30,000 and a chromatographic signal-to-noise (S/N) threshold of 1.5.

Retention time alignment was applied to correct for minor chromatographic shifts across runs, followed by peak picking and adduct grouping. In positive ionization mode, the following adducts were considered for feature consolidation: [M+H]^+^, [M+Na]^+^, [M+K]^+^, and [M+NH_4_]^+^. This step ensured that multiple ionic species originating from the same molecular entity were grouped into a single feature.

#### 4.5.6. Data Filtering and Quality Control

The feature-by-sample matrix generated by Compound Discoverer was subjected to a structured filtering strategy to enhance data reliability and reduce analytical noise. Background subtraction was applied to minimize contributions from solvent and system-derived signals. Features detected in fewer than 15% of the biological samples were removed by evaluating the gap status. To further improve data quality, features with a Peak Rating lower than 3.5 were excluded, as this parameter reflects poor chromatographic peak shape and reduced confidence in feature integration.

#### 4.5.7. Metabolite Annotation and Library Searching

Metabolite annotation was performed using an integrated, multi-tiered library search strategy implemented in Compound Discoverer 3.3. Putative compound assignments were generated by combining accurate mass measurements, isotopic pattern evaluation, and MS/MS spectral matching.

The workflow employed the Predict Composition, mzCloud, mzVault, and Mass Lists nodes for spectral-based annotation. In addition, the ChemSpider node was used to query multiple databases relevant to human biofluid metabolomics, including BioCyc, FooDB, the Human Metabolome Database (HMDB), the Kyoto Encyclopedia of Genes and Genomes (KEGG), LIPID MAPS, MassBank, NIST Spectra, and the Serum Metabolome Database. This comprehensive annotation strategy enabled consistent putative identification of metabolites across different extraction protocols and chromatographic platforms, supporting comparative evaluation of metabolomic coverage and analytical performance.

### 4.6. Evaluation of Key Steps in Metabolomics Analysis

Variability arising from key steps of the metabolomics workflow was systematically investigated, focusing on sample extraction, chromatographic separation, and blood matrix selection. Matrix-related differences in metabolomic profiling were evaluated using four biofluids: serum, plasma heparin, plasma EDTA, and plasma citrate. Each biological sample was analysed under two complementary chromatographic conditions: RP and HILIC column, in positive ionization mode.

To benchmark the analytical platform, two complementary reference strategies were adopted. The NIST SRM 1950 metabolite panel was used as a conceptual framework to assess the detection of 75 consensus metabolites representative of diverse chemical classes. In parallel, a certified yeast reference material was analysed to further evaluate analytical consistency and reproducibility across experimental conditions.

#### 4.6.1. Sample Extraction Evaluation

Three extraction protocols were compared by analysing the in-house pool of heparinised plasma. The extraction protocols consist of a monophasic protein precipitation by IPA [43], MeOH/ACN [44] and a modified Matyash biphasic extraction [21]. These three protocols were also applied on the unlabelled metabolite of certified yeast extract (Cambridge Isotope Laboratory, Cambridge, MA, USA) to evaluate the repeatability of the different sample extraction (Supplementary Materials). The samples were prepared and analysed in triplicate in each procedure.

#### 4.6.2. Matrix Effect

To evaluate the variability of four different biofluid matrices (serum, plasma heparin, plasma EDTA, and plasma citrate), each matrix was prepared according to extraction protocol A performed in triplicate, followed by HPLC-HRMS analysis with RP and HILIC setup.

To evaluate the effect of anticoagulants on ionization, six adducts were searched in Skyline for each molecule, based on their respective *m*/*z* values. For each compound, the following adducts were analysed: [M]^+^, [M+H]^+^, [M+Na]^+^, [M+K]^+^, [M+NH_4_]^+^, and [M−H_2_O+H]^+^. For glucose, the [M+Cl]^−^ adduct was additionally evaluated in negative ionization mode. Predominant adducts were identified based on relative signal intensity, and matrix-related differences were assessed descriptively.

#### 4.6.3. Targeted Reference Panel

To validate the analytical workflow, a targeted assessment was performed using two complementary reference strategies. First, the NIST SRM 1950 (Standard Reference Material^®^ 1950, Metabolites in Frozen Human Plasma, revision 12 October 2023) metabolite panel was employed as a conceptual benchmark. Specifically, the consensus list of 75 key compounds and their associated chemical classes was used to guide the recognition of corresponding *m*/*z* signatures within the experimental blood matrix datasets. This approach enabled the systematic categorization of detected features and provided a robust reference framework for evaluating detection rates and relative abundance patterns across different matrices. In parallel, a certified yeast reference material was physically analysed to assess extraction repeatability and precision for a defined set of known metabolites. Compounds reported in the manufacturer’s technical documentation were targeted within the LC–MS datasets to assess analytical precision and detection consistency.

Targeted peaks for these metabolites were integrated using Skyline software v. 24.1.0.414, based on MS1 full-scan data acquired at a resolution of 120,000 at 400 *m*/*z*. Variability assessment was performed only on the [M+H]^+^ adduct.

### 4.7. Statistical Analysis

Statistical analyses were performed to evaluate variability arising from key steps of the untargeted metabolomics workflow, including sample extraction strategy, chromatographic separation, and blood matrix selection. All univariate and multivariate analyses were conducted using the post-processing nodes implemented in Compound Discoverer version 3.3 (Thermo Fisher Scientific), unless otherwise specified.

#### 4.7.1. Multivariate Analysis

To assess the impact of different extraction strategies on global metabolomic profiles, unsupervised multivariate analyses were applied. Principal component analysis (PCA) was used to explore similarities and differences among samples prepared using protocols A, B, and C, including both the polar lower and MTBE-nonpolar upper phases generated by the biphasic extraction. PCA models were constructed independently for datasets acquired using RP and HILIC in order to evaluate how each chromatographic configuration influenced the discrimination of metabolomics signatures associated with the different extraction chemistries. PCA score plots were used to assess clustering consistency within triplicates and separation among experimental groups.

To further support the PCA results, two-way hierarchical clustering analysis was performed using normalized peak areas. Clustering was based on Euclidean distance and applied to visualize systematic similarities and differences in metabolite abundance patterns across extraction protocols, chromatographic modes, and blood matrices.

#### 4.7.2. Assessment of Analytical Repeatability

Intra-day analytical repeatability was evaluated using the CV% of peak areas calculated across triplicate analyses for each experimental condition. CV% calculations included all detected features, regardless of whether they were putatively annotated or remained unidentified, provided that the features were consistently detected in all three replicates of a given protocol.

Feature-level reproducibility was assessed using a CV% ≤ 30% threshold. This criterion was selected in accordance with established practices in untargeted HPLC–MS metabolomics, where a wide range of metabolite classes with heterogeneous physicochemical properties and concentration levels are analysed [45]. The proportion of features meeting this criterion was used as an indicator of analytical robustness across extraction protocols, chromatographic platforms, and blood matrices.

#### 4.7.3. Metabolite Coverage and Overlap Analysis

To evaluate metabolite coverage and the degree of overlap among experimental conditions, Venn diagram analyses were performed. Only putatively annotated metabolites consistently detected in 100% of triplicate analyses for each condition were included. Venn diagrams were used to compare shared and unique metabolites across extraction protocols, chromatographic modes, and blood matrices.

Comparisons were preferentially performed between experimental groups yielding the highest number of detected metabolites, as these datasets provide greater statistical robustness and minimize bias introduced by low-coverage conditions. This approach enabled meaningful assessment of protocol-dependent and matrix-dependent differences in metabolomic coverage while avoiding overinterpretation of sparsely populated feature sets.

#### 4.7.4. Targeted Panel Statistical Analysis

For target panel, evaluations encompassed different sample preparation protocols, matrix effects, and HPLC–HRMS conditions, with the coefficient of variation (CV%) applied as a quantitative indicator of reliability. Box plots were used to summarize CV% distributions across extraction protocols, chromatographic modes, and blood matrices.

#### 4.7.5. Data Visualization

Box plots and circular bar plots were generated using the ggplot2 package in the R statistical environment (version 4.3.2). These visualizations were used to support the interpretation of metabolite coverage, analytical reproducibility, and ionization behaviour across experimental conditions.

## Figures and Tables

**Table 1:** Predominant adduct formation across different biological matrices using reversed-phase (RP) and hydrophilic interaction (HILIC) chromatography column.

HPLC Column	Compound	Detected Adducts	Matrix-Related Observations
RP (ESI+)	Most compounds	[M+H]^+^	Comparable adduct distribution among serum, EDTA, citrate, and heparin plasma
	Tyrosine, phenylalanine, valine	[M+Na]^+^, [M]^+^	Higher sodium adduct contribution compared to other metabolites
	Creatinine, Valine	[M+H]^+^ [M+Na]^+^, [M+K]^+^	Increased [M+K]^+^ formation in EDTA vs. other matrices
	Methionine	[M+NH_4_]^+^	EDTA plasma showed increased [M+H]^+^ formation
	Arginine	[M+H]^+^ [M+Na]^+^, [M+K]^+^	No [M+K]^+^ adduct detected in EDTA plasma
	Lutein	[M+NH_4_]^+^	Different adduct composition in serum; not detected in heparin plasma
	Zeaxanthin, 4-pyridoxic acid, progesterone	[M+NH_4_]^+^	Similar adduct profiles across matrices
HILIC (ESI+)	Most compounds	[M+H]^+^	Limited matrix effect on adduct formation
	Tocopherol, lutein, zeaxanthin	[M]^+^, [M+NH_4_]^+^	Lutein and zeaxanthin showed 100% [M+Na]+ formation in serum only
	4-pyridoxic acid	[M]^+^, [M+NH_4_]^+^	Comparable adduct distribution among matrices
	Testosterone	[M+H]^+^, [M+Na]^+^	Increased sodium adduct in citrate plasma vs. other matrices
HILIC (ESI−)	Glucose	[M+Cl]^−^	Highest signal observed in negative ion mode

**Table 2:** Reversed-phase liquid chromatography solvent gradient. Phase B is 0.1% *v*/*v* formic acid, and phase C is 100% acetonitrile.

Time (min)	Phase B (%)	Phase C (%)
0	70	30
2	70	30
15	50	50
18	50	50
32	25	75
35	25	75
40	5	95
43	5	95
43	70	30
48	70	30

**Table 3:** Hydrophilic interaction chromatography solvent gradient. Phase A is acetate buffer 10 mM pH 6.6, and phase C is 100% acetonitrile.

Time (min)	A (%)	C (%)
0	20	80
2	20	80
10	35	65
12	35	65
20	50	50
23	20	80
25	20	80

## Data Availability

The original contributions presented in this study are included in the article/Supplementary Material. Further inquiries can be directed to the corresponding author.

## Supplementary Materials

The following supporting information can be downloaded at: https://www.mdpi.com/article/10.3390/molecules31050814/s1.

## Abbreviations

The following abbreviations are used in this manuscript:

ACN	acetonitrile
CV%	coefficient of variation percentage
EDTA	ethylenediaminetetraacetic acid
FA	formic acid
HPLC-HRMS	high-performance liquid chromatography–high-resolution mass spectrometry
HILIC	hydrophilic interaction chromatography
I.S.	internal standard
IPA	isopropyl alcohol
LLE	liquid–liquid extraction
MeOH	methanol
MTBE	methyl tert-butyl ether
PCA	principal component analysis
RP	reversed-phase
