# Supplementary material for: Investigating Variability in Metabolomics: A Comparative Study of Analytical Platforms and Blood Matrices Using HPLC-HRMS
Source: Molecules. 2026 Feb 28;31(5):814. doi: 10.3390/molecules31050814 (PMC12986168; doi:10.3390/molecules31050814)
Supplement: Supplementary file 1 [file molecules-31-00814-s001.zip › Supplementary.pdf]

Investigating Variability in Metabolomics: A Comparative Study of Analytical Platforms and Blood Matrices Using LC-HRMS

Giulia Guerra [1], Alessio Polymeropoulos [2], Elisabetta Venturelli [3,*], Veronica Huber [4], Francesco Segrado[3], Daniele Morelli [5] and Sabina Sieri [1]

1 Epidemiology and Prevention Unit, Fondazione IRCCS Istituto Nazionale dei Tumori di Milano, 20133 Milan, Italy; giulia.guerra@istitutotumori.mi.it (G.G.) https://orcid.org/0009-0003-4546-9718; sabina.sieri@istitutotumori.mi.it (S.S.) https://orcid.org/0000-0001-5201-172X

2 Biostatistics for Clinical Research, Fondazione IRCCS Istituto Nazionale dei Tumori di Milano, 20133 Milan, Italy; alessio.polymeropoulos@istitutotumori.mi.it https://orcid.org/0000-0002-2652-3964

3 Nutrition Research and Metabolomics Unit, Fondazione IRCCS Istituto Nazionale dei Tumori di Milano, 20133 Milan, Italy; francesco.segrado@istitutotumori.mi.it https://orcid.org/0000-0002-1270-4609

4 Unit of Translational Immunology, Fondazione IRCCS Istituto Nazionale dei Tumori di Milano, 20133 Milan, Italy; veronica.huber@istitutotumori.mi.it https://orcid.org/0000-0001-6304-3575

5 Laboratory Medicine Department, Fondazione IRCCS Istituto Nazionale dei Tumori di Milano, 20133 Milan, Italy; daniele.morelli@istitutotumori.mi.it https://orcid.org/0000-0002-1823-3764

* Correspondence: elisabetta.venturelli@istitutotumori.mi.it; Tel.: +39-02-2390-3745 https://orcid.org/0000-0002-7427-7032

Supplementary

ANALYSIS OF YEAST EXTRACT

Material

Unlabeled metabolite yeast extract ISO1-UNL lot. N. 20220615 2022.06.15 was purchased from Cambridge Isotope Laboratory (Tewksbury, MA).

Analytical procedure.

The extract was reconstituted with 2 mL of H_2O:MeOH 1:1 (v/v), vortexed for 5 minutes, and centrifuged at 20°C for 5 minutes at 3750 rpm. The supernatant was collected and diluted 1:10 with a solution of H_2O:MeOH 1:1 (v/v). The diluted working extract was subjected to three different sample extraction procedures, and each extracted sample was analyzed in triplicate by HPLC–HRMS. The three extraction protocols (A, B, and C) are described in detail in Section *4.4. Sample extraction* of Material and Methods, of the manuscript. Briefly, the protocols consisted of: protocol A, liquid–liquid extraction (LLE) with isopropanol (IPA); protocol B, LLE with a methanol/acetonitrile (MeOH/ACN) mixture; and protocol C, modified Matyash extraction, yielding two phases (polar lower phase and MTBE nonpolar upper phase).
Raw data were pre-processed using Skyline software.

Results

Sample extraction protocols

Single-phase extractions (Protocols A and B) yielded the most comprehensive results, with Protocol B leading in RP mode (68 compounds) and Protocol A in HILIC mode (65 compounds). In contrast, the biphasic Matyash extraction (Protocol C) required combining both phases to match this coverage, as the lower phase alone was notably limited, particularly in HILIC mode (13 compounds) (Table S1). Ultimately, Protocol A and Protocol B are the most efficient for high-throughput screening, offering maximal metabolite coverage in a single step without the need for multi-phase analysis.

Table S1 *Number of detected compounds among the 91 certified metabolites detected by reversed phase column (RP), and by HILIC column (HILIC). Protocols evaluated include: A, LLE extraction with isopropanol (IPA); B, LLE extraction with a methanol/acetonitrile (MeOH/ACN) mixture; and C, modified Matyash extraction (two phases: polar lower phase and MTBE-nonpolar upper phase)*

	**Protocol A**	**Protocol B**	**Protocol_C_Upper**	**Protocol_C_Lower**
**AREA CV% RP**	64	68	59	22
**AREA CV% HILIC**	65	60	58	13

Targeted Yeast Reference Panel: Detection Rates and Reproducibility

Analytical Reproducibility and Extraction Efficiency

The precision of the extraction protocols was evaluated by comparing the distribution of the coefficient of variation (CV%) for the yeast extract panel across both Reversed Phase (RP) and HILIC chromatographic platforms. A targeted assessment was performed using a defined set of known metabolites. The molecules were selected from the manufacturer's certificate of analysis. Molecule names and classifications were chosen according to this document.

For the certified yeast reference material (91 compounds), RP analysis yielded a detection rates of 64/91 compounds with protocol A, 68/91 with protocol B, 59/91 with protocol C_lower, and 22/91 with protocol C_upper, with corresponding median CV% of 4.9%, 8.5%, 21.9%, and 85.1%, respectively. Under HILIC analysis metabolite coverage remained comparable for protocols A, B, and C_lower (65/91, 60/91, and 58/91 compounds, respectively) but dropped to 13/91 for protocol C_nonpolar upper phase. The associated median CV% values were 29.8%, 24.0%, and 15.0% for the first three protocols, whereas protocol C_ nonpolar upper phase again exhibited poor analytical precision with a median CV% of 59.5% (Figure S1).

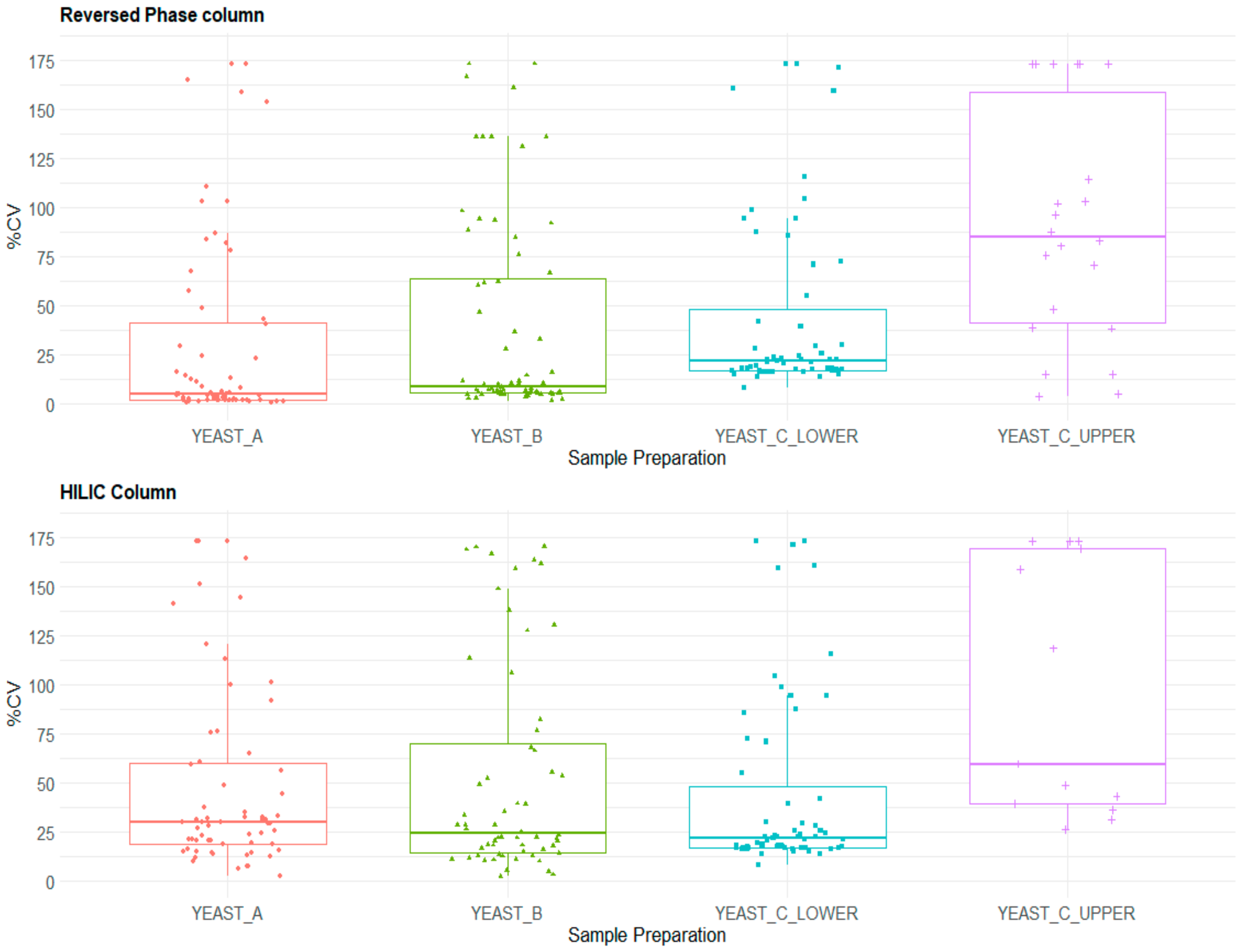

Figure S1. Box plot comparisons of coefficient of variation (%CV) distribution for compounds included in the yeast extract panel for each extraction protocol: protocol A, LLE extraction with isopropanol (IPA); protocol B, LLE extraction with a methanol/acetonitrile (MeOH/ACN) mixture; and protocol C, modified Matyash extraction (two phases: polar lower phase and MTBE-nonpolar upper phase). Data were acquired in positive ionization mode: (a) HPLC

analysis on a reversed-phase (RP) column; (b) HPLC analysis on a hydrophilic interaction liquid chromatography (HILIC) column.

Targeted Yeast Reference Panel: Detection Rates and Reproducibility

The metabolomic profiling of the yeast extract using Protocol A (IPA) demonstrated extensive coverage across both chromatographic platforms, with molecules categorized into amino acids and derivatives, fatty acids, hormones, and metabolites.

The reversed-phase (RP) analysis (Figure S2a) proved highly effective for the detection of the yeast-derived lipid fraction and non-polar metabolites. High signal intensities, expressed as log10 median peak areas, were observed for several amino acids, including alanine, isoleucine, and leucine, with values consistently exceeding 7.0. Furthermore, the RP platform successfully captured a wide range of fatty acids, such as docosahexaenoic acid (DHA), eicosapentaenoic acid (EPA), and arachidonic acid, alongside other key markers like uric acid and creatinine.

The HILIC analysis (Figure S2b) provided a complementary metabolic signature, characterized by an expanded detection of polar species. While maintaining high signal intensities for primary amino acids like alanine and isoleucine, the HILIC mode uniquely highlighted additional polar compounds, including serine and threonine. Notably, the hormonal profile in the yeast extract was extended in HILIC mode to include both cortisol and testosterone. However, in contrast to the RP results, the fatty acid coverage was significantly reduced, with only a few species such as lignoceric and linoleic acids reaching detectable levels.

Overall, the combination of Protocol A with both RP and HILIC columns ensures a comprehensive characterization of the yeast reference material, balancing the high-intensity detection of amino acids with specific coverage for lipids and polar hormones.

Note on Metabolite Classification and Nomenclature

The classification and naming of metabolites presented in this study (including the grouping of GSH/GSSG, mevalonic acid, and α-ketoisovalerate) were strictly aligned with the Certificate of Analysis (CoA) and official documentation provided by Cambridge Isotope Laboratories (CIL).

While alternative biochemical classifications (e.g., grouping glutathione under amino acids or α-ketoisovalerate under organic acids) are scientifically valid, the adoption of the supplier's original nomenclature was prioritized to ensure inter-laboratory comparability and adherence to certified reference standards. This approach minimizes ambiguity when benchmarking results against the Certified Yeast Reference Material used in this workflow.

In the interest of figure readability, abbreviated names derived from the CIL documentation were utilized. A comprehensive list of all abbreviations and their corresponding full chemical names is provided in the "Supplementary Retention Time" file.

(A)

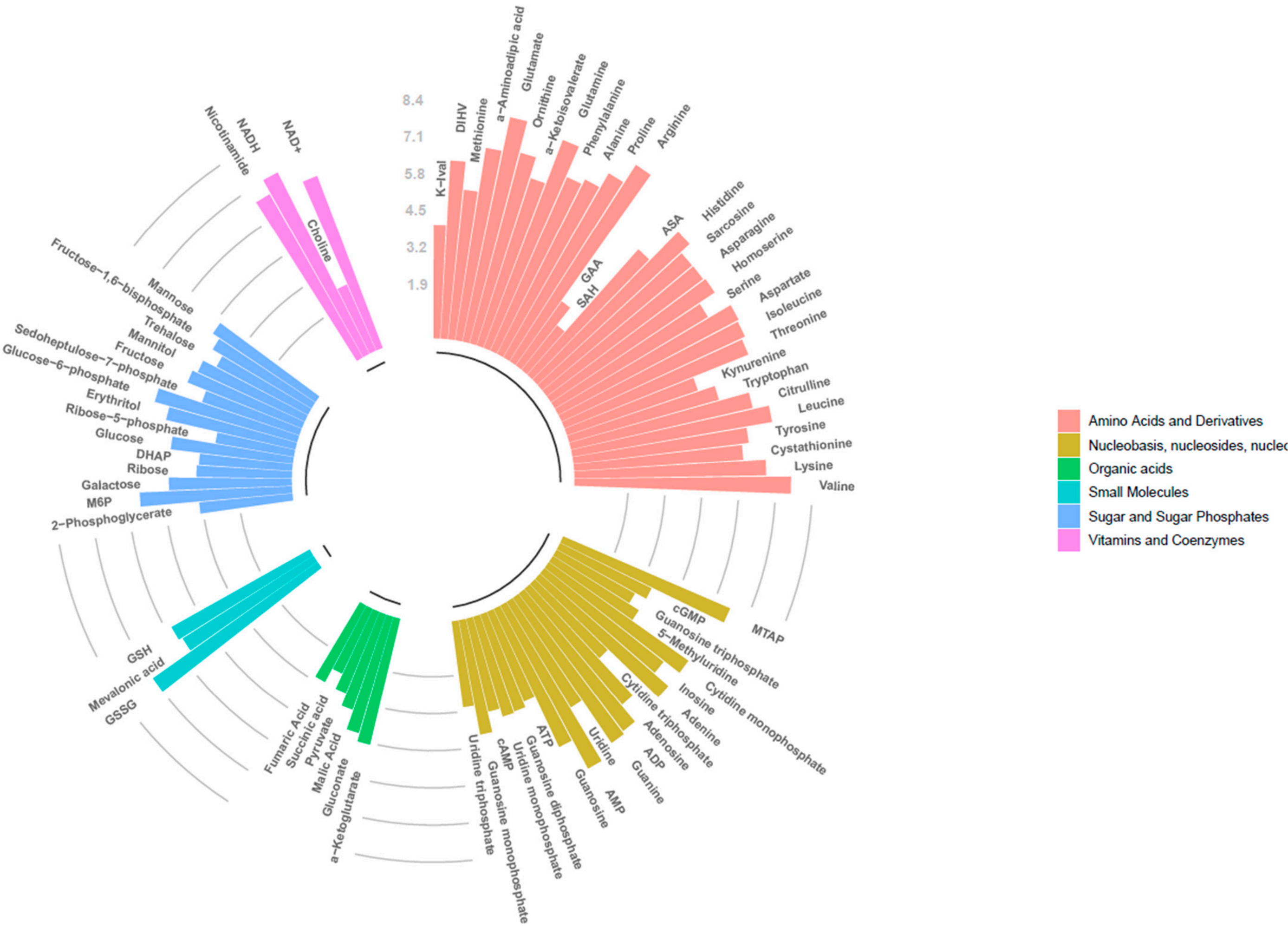
8.4
7.1
5.8
4.5
3.2
1.9
K−Ival
DIHV
Methionine
a−Aminoadipic acid
Glutamate
Ornithine
a−Ketoisovalerate
Glutamine
Phenylalanine
Alanine
Proline
Arginine
ASA
Histidine
Sarcosine
Asparagine
Homoserine
GAA
SAH
Serine
Aspartate
Isoleucine
Threonine
Kynurenine
Tryptophan
Citrulline
Leucine
Tyrosine
Cystathionine
Lysine
Valine
MTAP
cGMP
Guanosine triphosphate
5−Methyluridine
Cytidine monophosphate
Inosine
Adenine
Cytidine triphosphate
Adenosine
ADP
Guanine
Uridine
AMP
Guanosine
ATP
Guanosine diphosphate
Uridine monophosphate
cAMP
Guanosine monophosphate
Uridine triphosphate
Fumaric Acid
Succinic acid
Pyruvate
Malic Acid
Gluconate
a−Ketoglutarate
GSH
Mevalonic acid
GSSG
2−Phosphoglycerate
M6P
Galactose
Ribose
DHAP
Glucose
Ribose−5−phosphate
Erythritol
Glucose−6−phosphate
Sedoheptulose−7−phosphate
Fructose
Mannitol
Trehalose
Fructose−1,6−bisphosphate
Mannose
Nicotinamide
NADH
NAD+
Choline
Amino Acids and Derivatives
Nucleobasis, nucleosides, nucle
Organic acids
Small Molecules
Sugar and Sugar Phosphates
Vitamins and Coenzymes

(B)

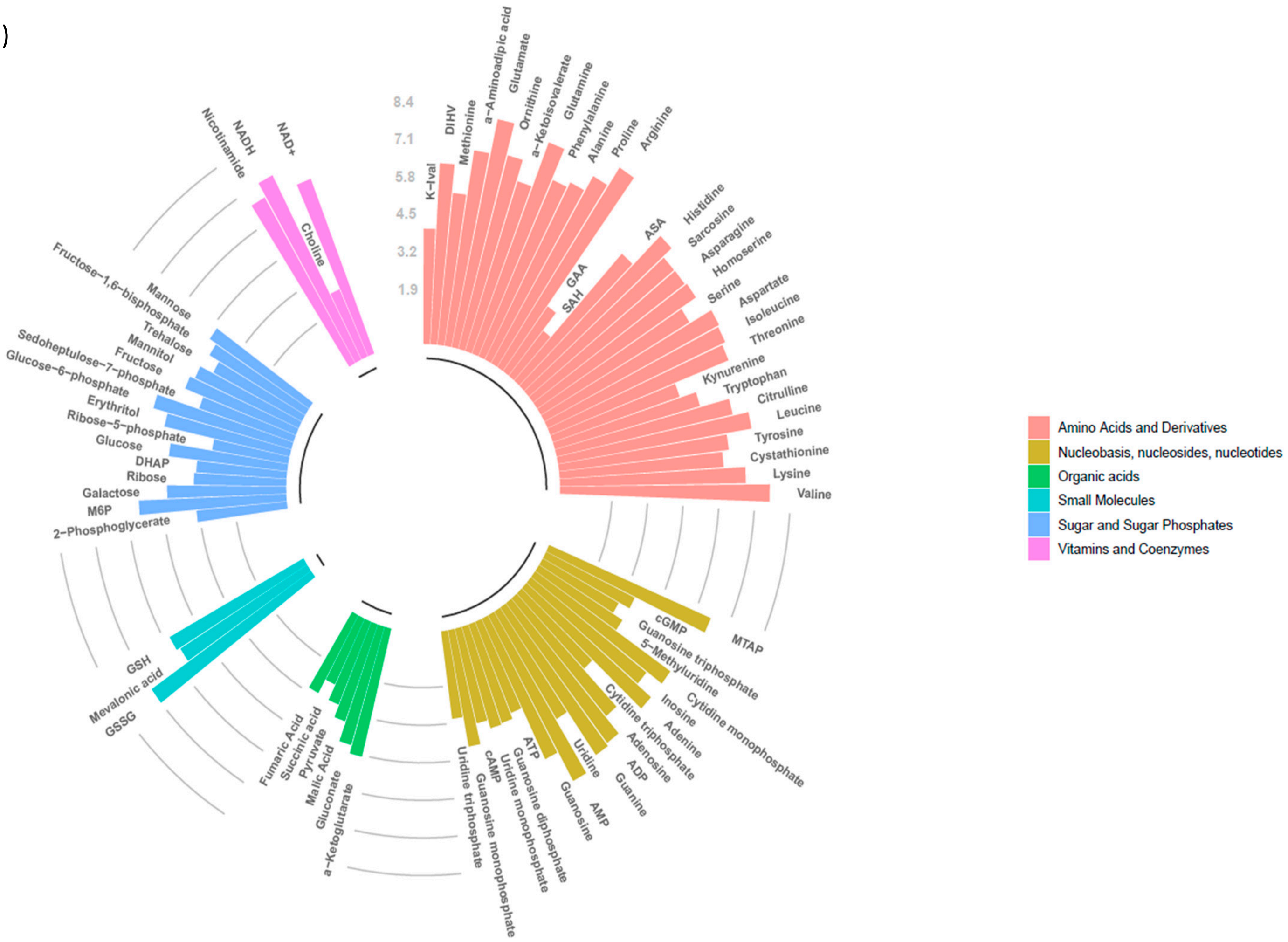

Figure S2. Circular bar plot showing molecules detected in the yeast extract using Protocol A, expressed as $\log_{10}$ median peak area. Data were acquired in positive ionization mode: (a) HPLC analysis on a reversed-phase (RP) column; (b) HPLC analysis on a hydrophilic interaction liquid chromatography (HILIC) column.

Compound names are abbreviated. See the Supplementary Retention Time file for full nomenclature and additional information.

Compound Discoverer processing nodes

Bioinformatics pipeline for metabolite identification and statistical processing.

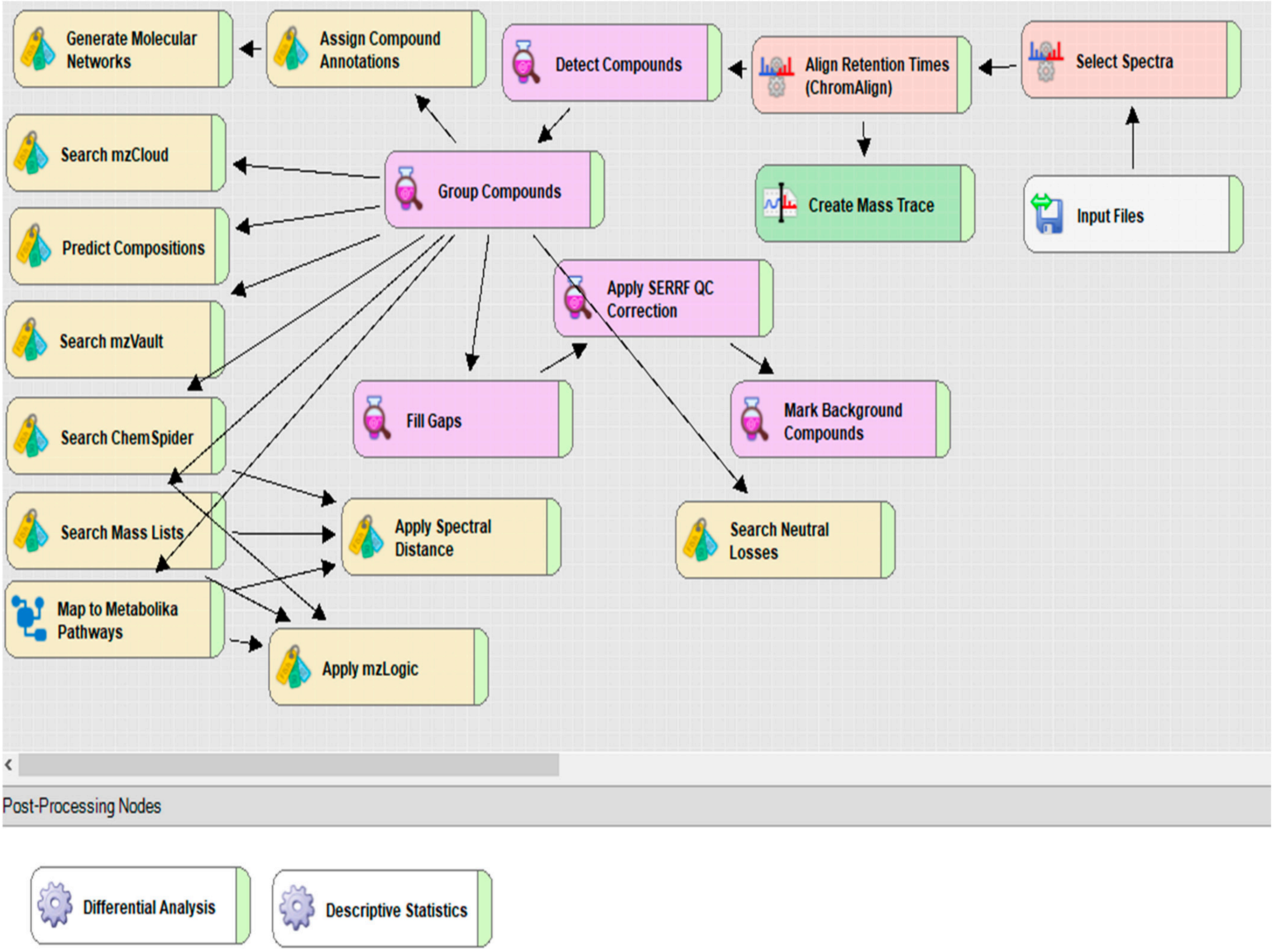

Figure S3. Workflow of the processing and analysis of raw data by Compound Discoverer 3.3 software for the peak detection and identification.

Sample extraction protocols

Venn Analysis of Extraction Efficiency and Column Compatibility for Plasma Heparin Metabolites

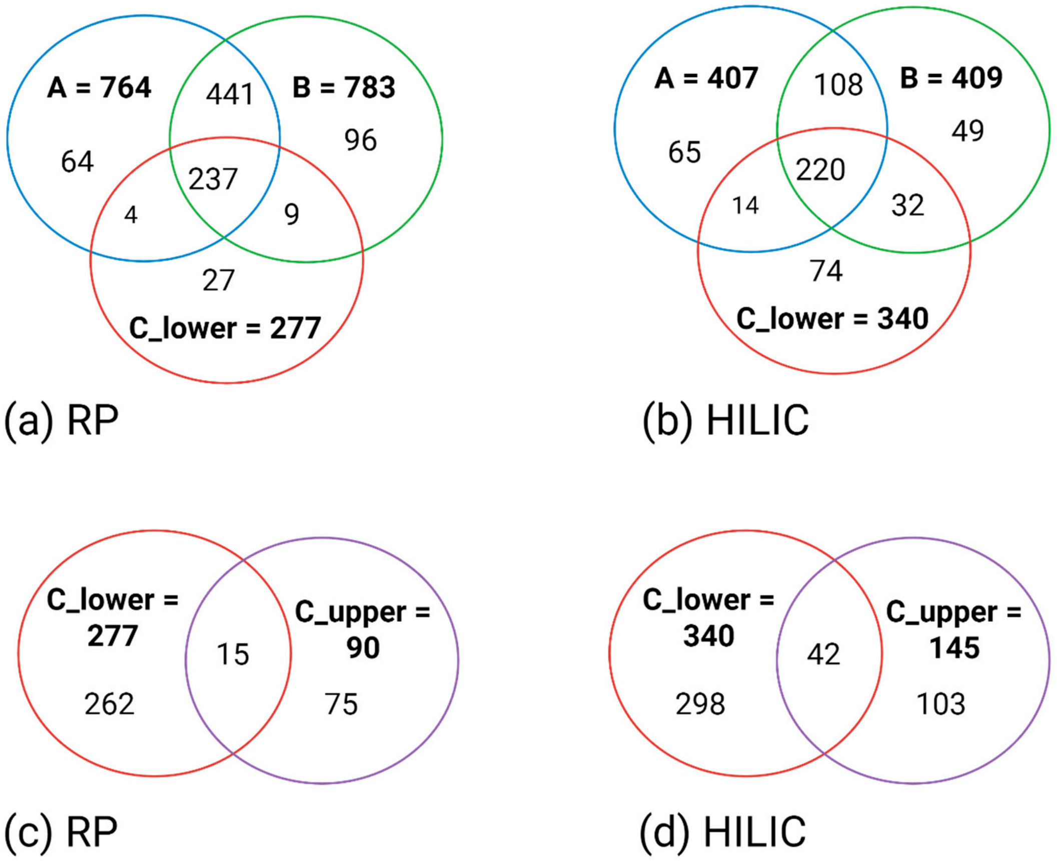

Figure S4. Venn diagram of metabolites detected in 100% of triplicates for each plasma heparin extraction protocol and putatively identified using Compound Discoverer.

(a) and (b) Comparison of metabolites extracted with protocols A (LLE with IPA), B (LLE with MeOH/ACN), and C (modified Matyash: polar lower phase and nonpolar upper phase), analyzed in positive ionization mode on RP (a) or HILIC (b) columns.

(c) and (d) Comparison of metabolites in protocol C lower versus upper phase, analyzed in positive ionization mode on RP (c) or HILIC (d) columns.

Analytical Reproducibility according sample protocols of Plasma heparin Features in RP and HILIC Platforms

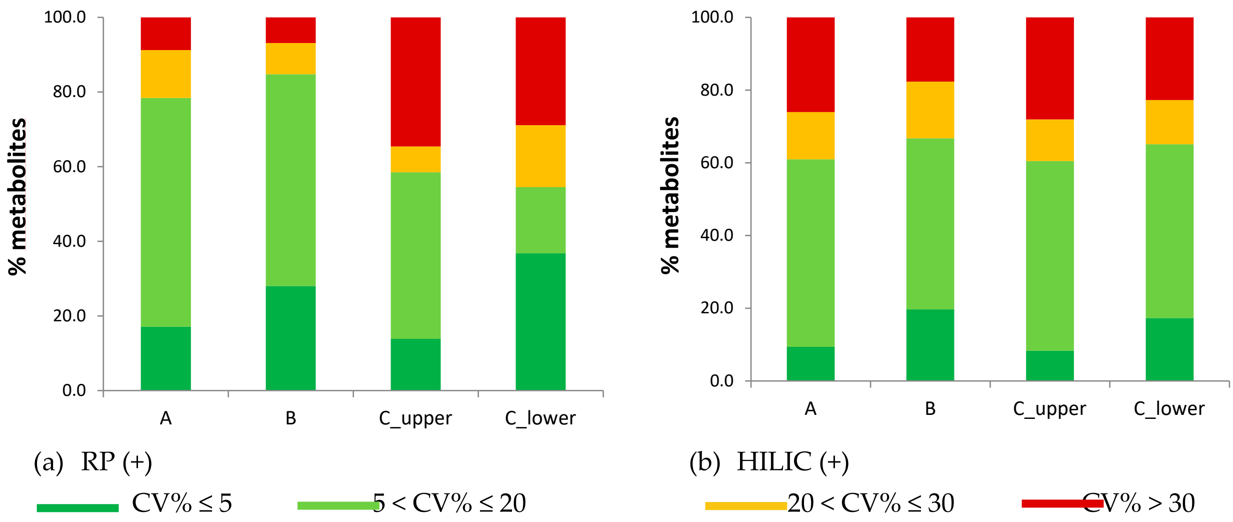

Figure S5. Percentage of features with a coefficient of variation minus of 5% (dark green), between 5% and 20 % (light green), between 20% and 30% (yellow) and more than 30% (red). The selected features were detected at 100% in all triplicate for each protocol of plasma heparin extraction. Protocol A, LLE extraction by IPA; protocol B, LLE extraction by MeOH/ACN mixture; protocol C, modified Matyash extraction (two phases: polar lower phase and MTBE-nonpolar upper phase). Data were acquired in positive ionization mode: (a) HPLC analysis on a reversed-phase (RP) column; (b) HPLC analysis on a hydrophilic interaction liquid chromatography (HILIC) column.

Table S2. Quantitative evaluation of the variability in plasma heparin samples due to the selection of the sample preparation protocol, column and acquisition parameter settings. The percentage of the number of the metabolites with a CV% ≤ 30 for each setting was selected as the parameter that express the variability evaluation

	**RP**	**HILIC**	
	**% Metabolites with CV% ≤ 30**	**%Metabolites with CV% ≤ 30**	**AVERAGE (%)**
**Protocol A**	91.2	73.9	82.6
**Protocol B**	93.1	82.3	87.8
**Protocol C_upper**	65.4	71.9	68.65
**Protocol C_lower**	71.1	77.2	73.7
**AVERAGE (%)**	80.2	76.3	

Protocol A= LLE extraction by IPA; protocol B= LLE extraction by MeOH/ACN mixture; protocol C= modified Matyash extraction (two phases: polar lower phase and MTBE-nonpolar upper phase)

Blood matrix comparison

Venn Analysis of blood matrix comparison according RP and HILIC chromatographic conditions

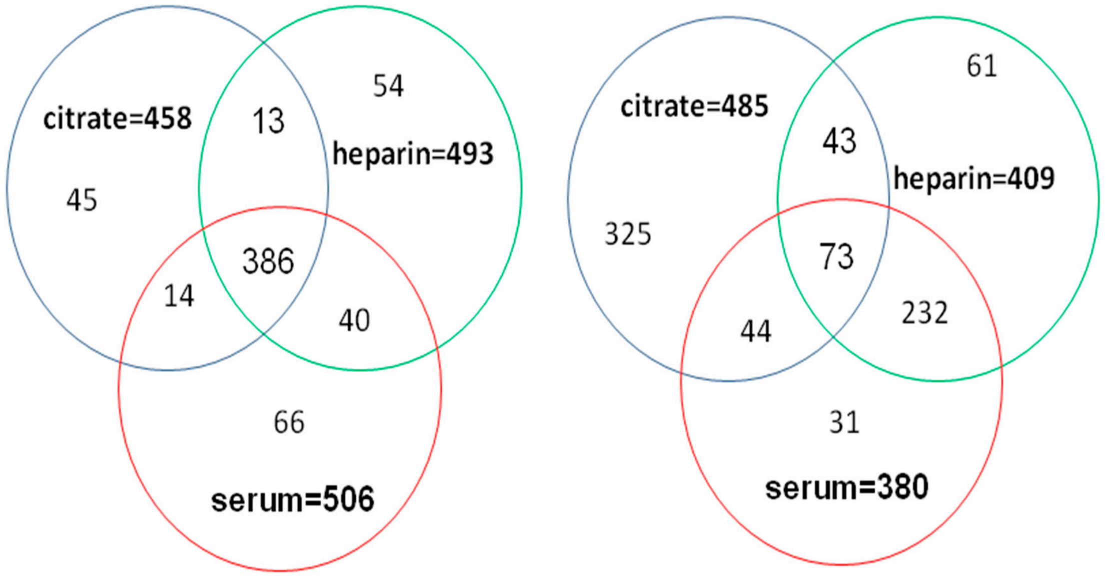

(a) RP (+)   (b) HILIC (+)

Figure S6. Venn diagram of the metabolites detected at 100% in all the triplicates for each matrix and whose putative name is identified by Compound Discoverer. Comparation of the metabolites extracted by protocol A in plasma heparin, plasma citrate and serum. Data were acquired in positive ionization mode: (a) HPLC analysis on a reversed-phase (RP) column; (b) HPLC analysis on a hydrophilic interaction liquid chromatography (HILIC) column.

Analytical Reproducibility according blood matrix samples Features in RP and HILIC Platforms

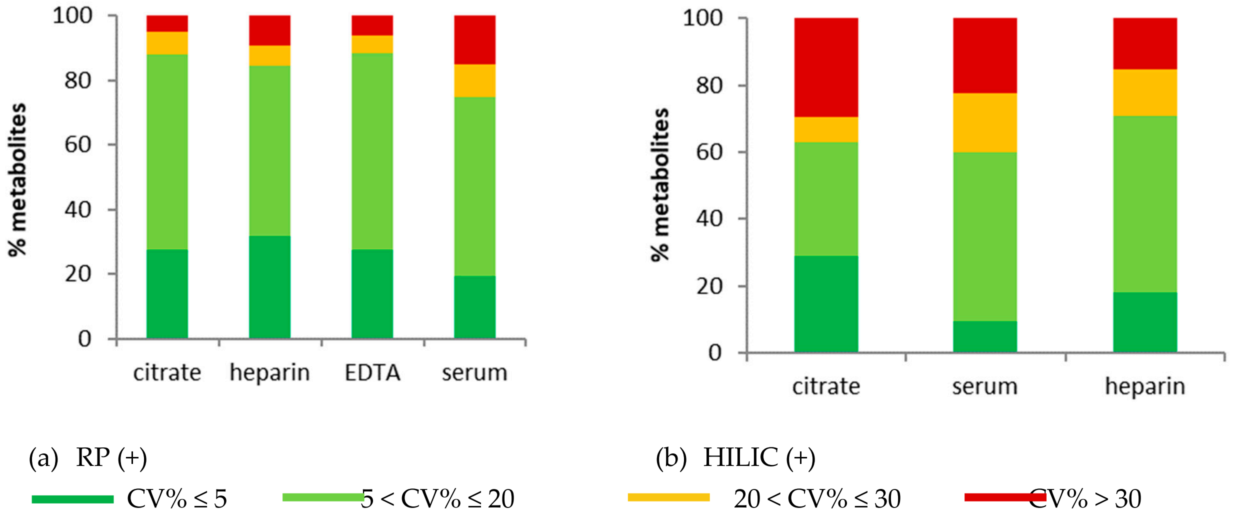

(a) RP (+)   (b) HILIC (+)

Figure S7. Percentage of features with a coefficient of variation minus of 5% (dark green), between 5% and 20 % (light green), between 20% and 30% (yellow) and more than 30% (red). The selected features were detected at 100% in triplicates for each matrix. Data were acquired in positive ionization mode: (a) HPLC analysis on a reversed-phase (RP) column; (b) HPLC analysis on a hydrophilic interaction liquid chromatography (HILIC) column.

Table S3. Quantitative assessment of metabolite variability as a function of biofluid matrix and chromatographic separation. Variability was evaluated as the percentage of metabolites with a coefficient of variation (CV%) ≤ 30 for each analytical setting (RP+ and HILIC+). Average values across chromatographic conditions and matrices are reported.

	**RP +**	**HILIC +**	
	**% Metabolites with CV% ≤ 30**	**% Metabolites with CV% ≤ 30**	**AVERAGE (%)**
**Plasma citrate**	95.0	70.5	82.8
**Plasma heparin**	90.7	77.7	84.2
**Plasma EDTA**	94.0	n.d.	94
**Serum**	85.1	84.6	84.9
**AVERAGE (%)**	91.2	77.6	

Table S4. Average retention time (RT), recovery, and coefficient of variation (CV) of the internal standard choline-d9 ([M+H]$^+$, m/z 113.164) across different extraction protocols in human heparinized plasma and yeast, and across four biofluid matrices extracted using Protocol B.

	RP+ column			HILIC+ column		
	**Mean RT (min)**	**Mean recovery (%)**	**Recovery CV (%)**	**Average RT (min)**	**Mean recovery (%)**	**Recovery CV (%)**
**Solvent (I.S.)**	0.5	-	-	3.9	-	-
**Yeast protocol A**	0.49	191.9	2.7	3.91	137.83	1.35
**Yeast protocol B**	0.49	134.8	5.1	3.93	123.39	2.09
**Yeast protocol C_upper**	0.49	1.1	6.3	3.92	2.11	6.17
**Yeast protocol C_lower**	0.49	96.0	13.8	3.92	89.90	9.34
**Plasma protocol A**	0.60	191.9	2.7	3.18	137.83	1.35
**Plasma protocol B**	0.60	89.8	< 1	3.17	99.1	1.1
**Plasma protocol C_upper**	0.50	1.2	21.6	3.91	2.5	29.7
**Plasma protocol C_lower**	0.55	31.2	6.3	3.17	56.7	3.3
**Plasma heparin**	0.61	51.2	1.75	3.16	100	3.2
**Plasma citrate**	0.59	42.6	6.52	3.16	101	3.4
**Plasma EDTA**	0.59	37	n.d.	n.d.	n.d.	n.d.
**Serum**	0.59	45.8	9.37	3.14	97	5.1

Protocol A= LLE extraction by IPA; protocol B= LLE extraction by MeOH/ACN mixture; protocol C= modified Matyash extraction (two phases: polar lower phase and MTBE-nonpolar upper phase)
