# Supplementary material for: Investigating Variability in Metabolomics: A Comparative Study of Analytical Platforms and Blood Matrices Using HPLC-HRMS
Source: Molecules. 2026 Feb 28;31(5):814. doi: 10.3390/molecules31050814 (PMC12986168; doi:10.3390/molecules31050814)
Supplement: Supplementary file 1 [file molecules-31-00814-s001.zip › Supplementary_AdductReversedPhase.pdf]

Investigating Variability in Metabolomics: A Comparative Study of Analytical Platforms and Blood Matrices Using LC-HRMS

Giulia Guerra [1], Alessio Polymeropoulos [2], Elisabetta Venturelli [3,*], Veronica Huber [4], Francesco Segrado[3], Daniele Morelli [5] and Sabina Sieri [1]

1 Epidemiology and Prevention Unit, Fondazione IRCCS Istituto Nazionale dei Tumori di Milano, 20133 Milan, Italy; giulia.guerra@istitutotumori.mi.it (G.G.) https://orcid.org/0009-0003-4546-9718; sabina.sieri@istitutotumori.mi.it (S.S.) https://orcid.org/0000-0001-5201-172X

2 Biostatistics for Clinical Research, Fondazione IRCCS Istituto Nazionale dei Tumori di Milano, 20133 Milan, Italy; alessio.polymeropoulos@istitutotumori.mi.it https://orcid.org/0000-0002-2652-3964

3 Nutrition Research and Metabolomics Unit, Fondazione IRCCS Istituto Nazionale dei Tumori di Milano, 20133 Milan, Italy; francesco.segrado@istitutotumori.mi.it https://orcid.org/0000-0002-1270-4609

4 Unit of Translational Immunology, Fondazione IRCCS Istituto Nazionale dei Tumori di Milano, 20133 Milan, Italy; veronica.huber@istitutotumori.mi.it https://orcid.org/0000-0001-6304-3575

5 Laboratory Medicine Department, Fondazione IRCCS Istituto Nazionale dei Tumori di Milano, 20133 Milan, Italy; daniele.morelli@istitutotumori.mi.it https://orcid.org/0000-0002-1823-3764

* Correspondence: elisabetta.venturelli@istitutotumori.mi.it; Tel.: +39-02-2390-3745 https://orcid.org/0000-0002-7427-7032

Comparative histogram of the six monitored adducts for the SRM compound list in the RP column for the three matrices. Values are expressed as relative percentages of the peak area intensity.

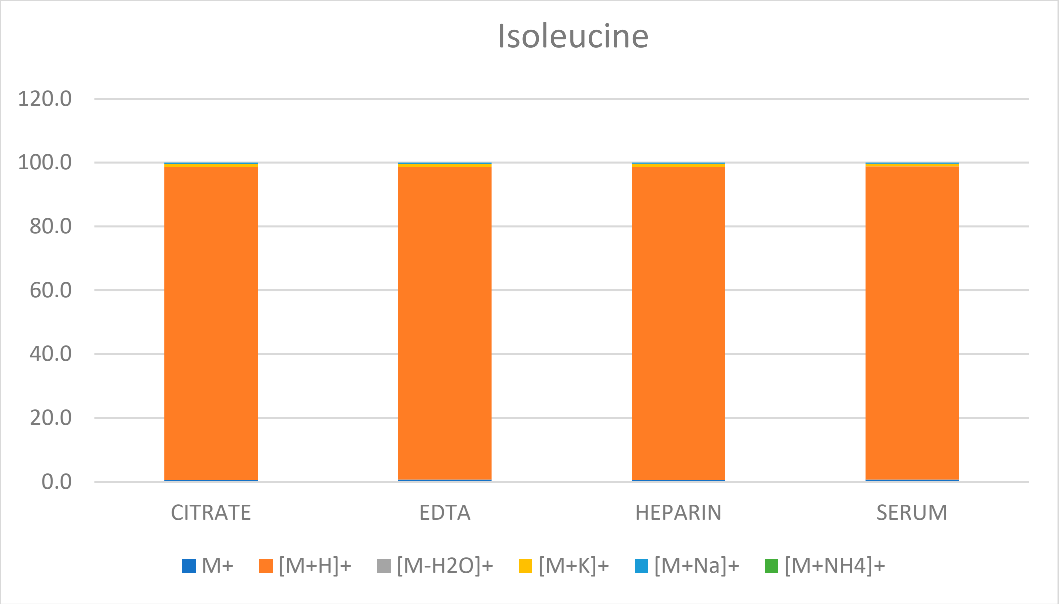

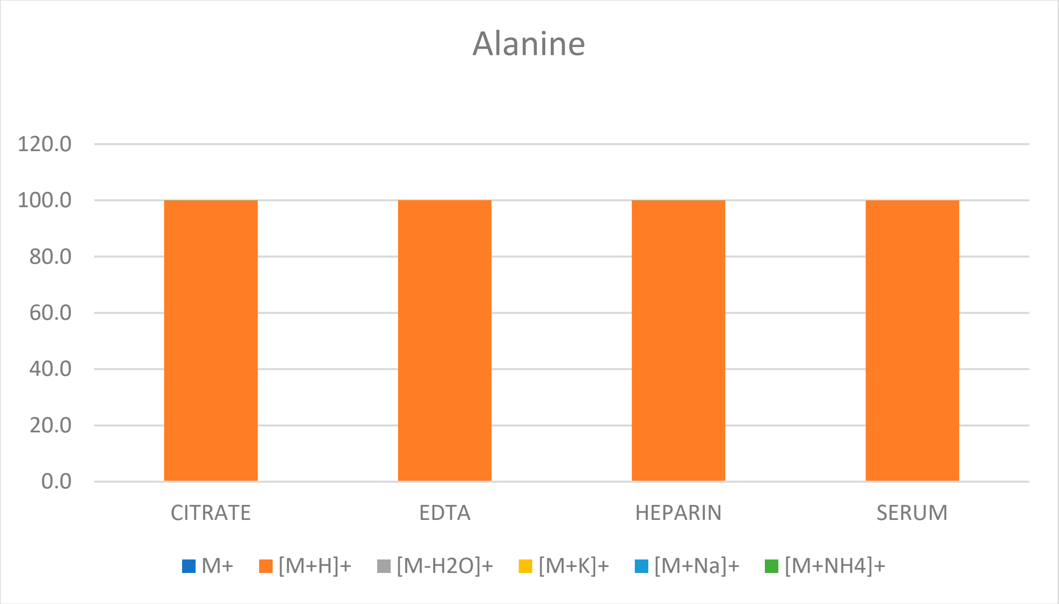

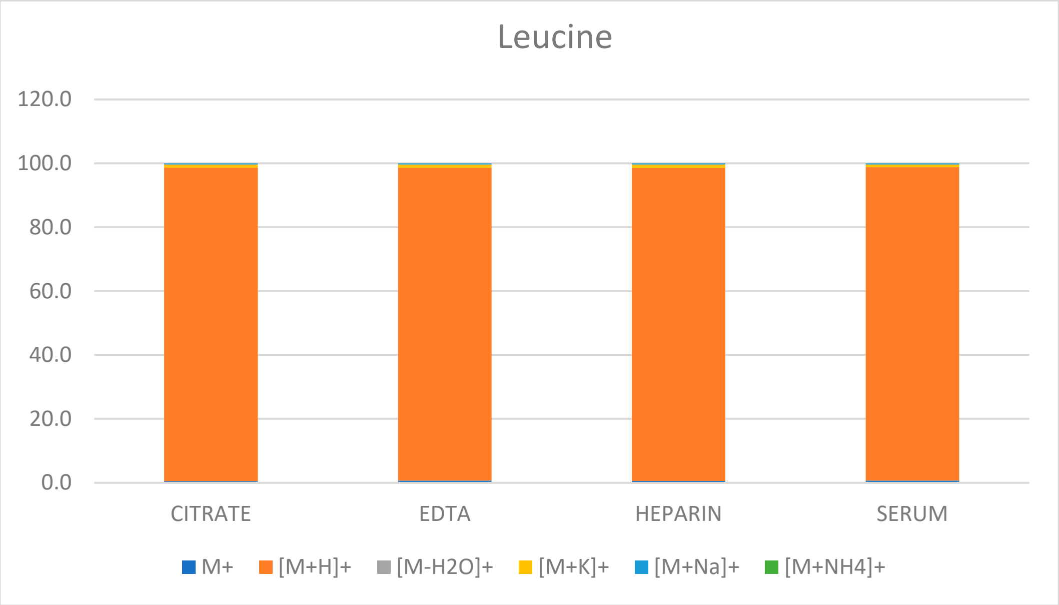
Leucine
120.0
100.0
80.0
60.0
40.0
20.0
0.0
CITRATE
EDTA
HEPARIN
SERUM
M+
[M+H]+
[M-H2O]+
[M+K]+
[M+Na]+
[M+NH4]+

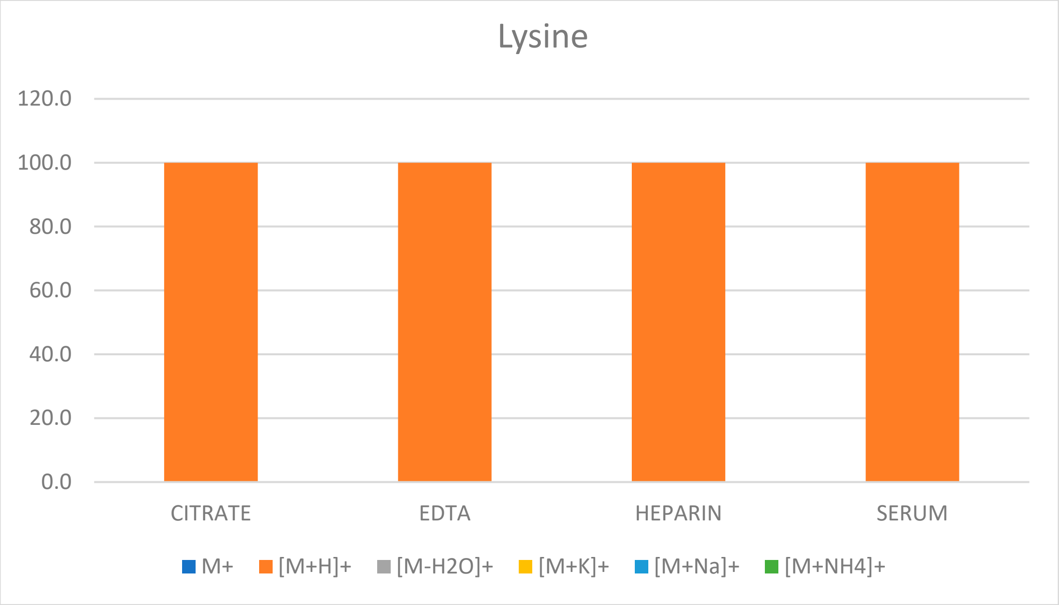
Lysine
120.0
100.0
80.0
60.0
40.0
20.0
0.0
CITRATE
EDTA
HEPARIN
SERUM
M+
[M+H]+
[M-H2O]+
[M+K]+
[M+Na]+
[M+NH4]+

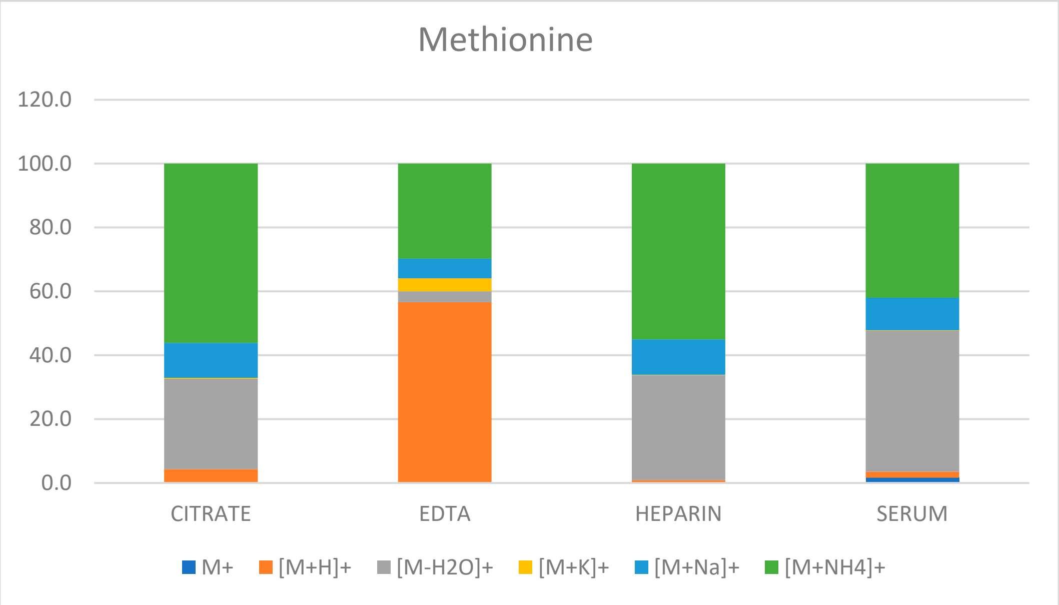
Methionine
120.0
100.0
80.0
60.0
40.0
20.0
0.0
CITRATE
EDTA
HEPARIN
SERUM
M+
[M+H]+
[M-H2O]+
[M+K]+
[M+Na]+
[M+NH4]+

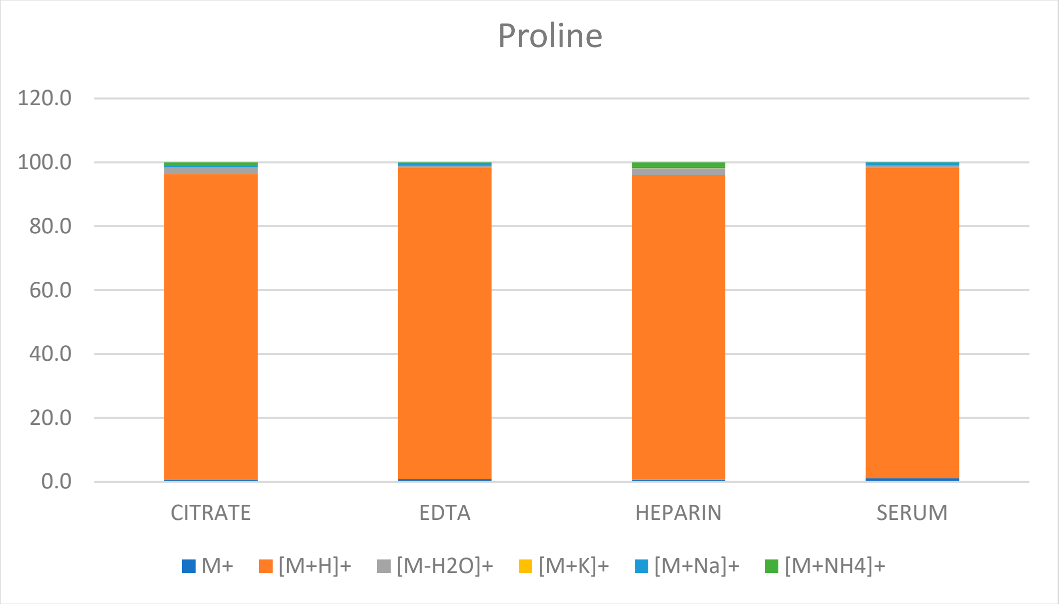
Proline
120.0
100.0
80.0
60.0
40.0
20.0
0.0
CITRATE
EDTA
HEPARIN
SERUM
M+
[M+H]+
[M-H2O]+
[M+K]+
[M+Na]+
[M+NH4]+

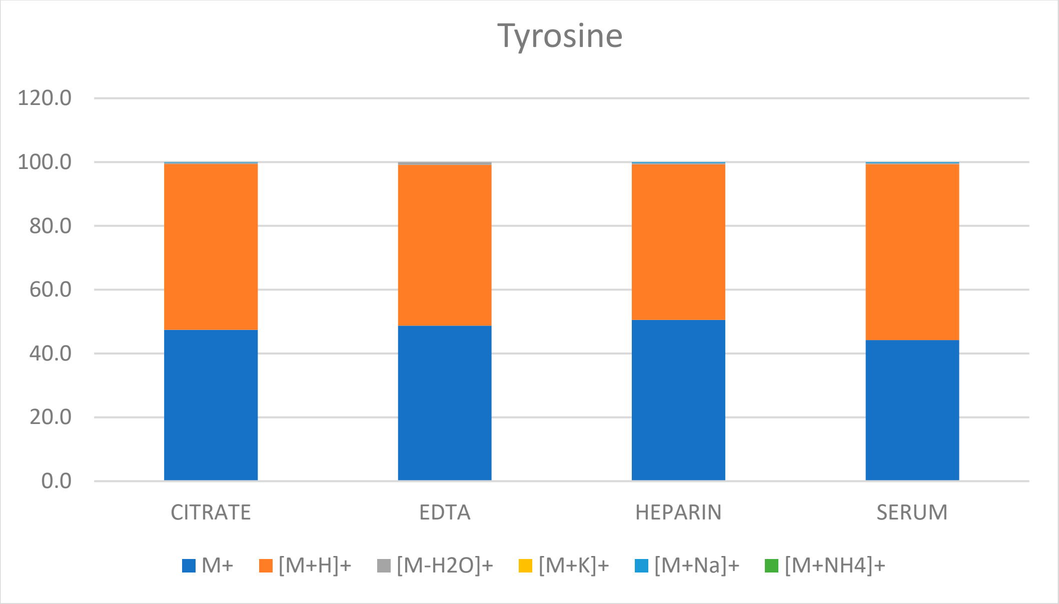
Tyrosine
120.0
100.0
80.0
60.0
40.0
20.0
0.0
CITRATE
EDTA
HEPARIN
SERUM
M+
[M+H]+
[M-H2O]+
[M+K]+
[M+Na]+
[M+NH4]+

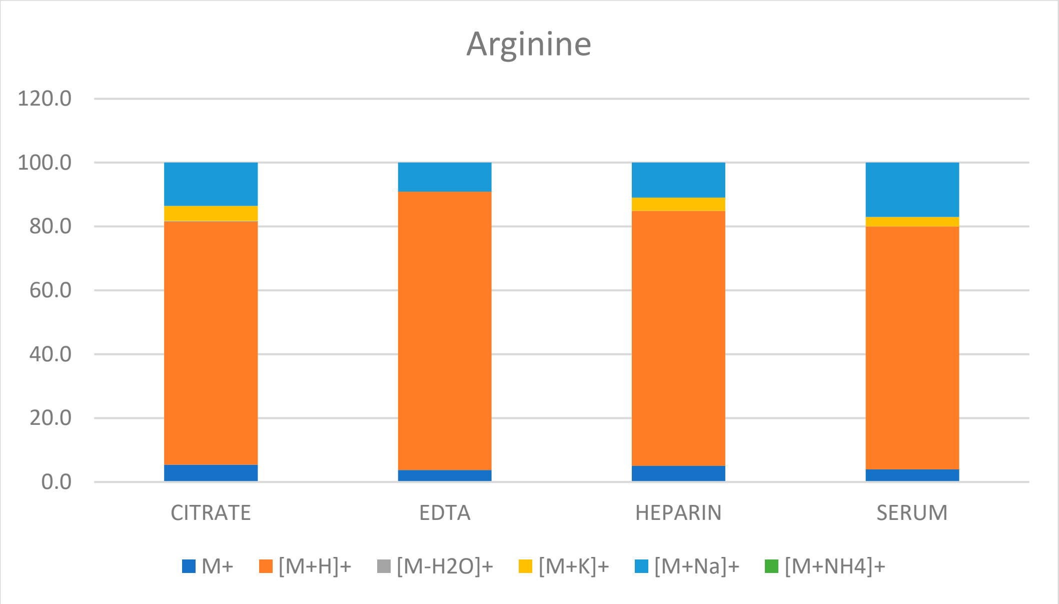
Arginine
120.0
100.0
80.0
60.0
40.0
20.0
0.0
CITRATE
EDTA
HEPARIN
SERUM
M+
[M+H]+
[M-H2O]+
[M+K]+
[M+Na]+
[M+NH4]+

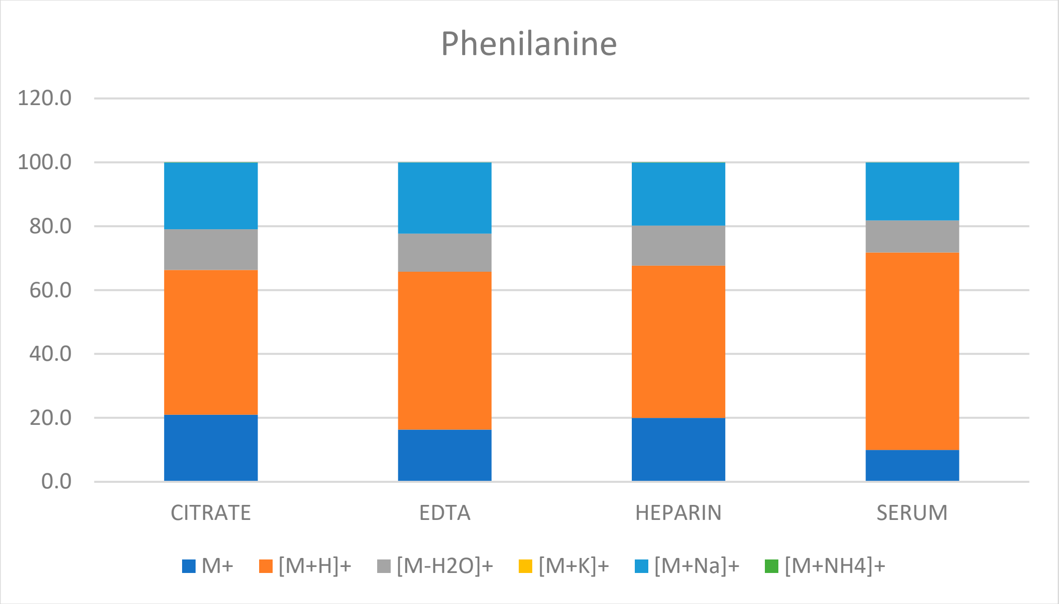
Phenilanine
120.0
100.0
80.0
60.0
40.0
20.0
0.0
CITRATE
EDTA
HEPARIN
SERUM
M+
[M+H]+
[M-H2O]+
[M+K]+
[M+Na]+
[M+NH4]+

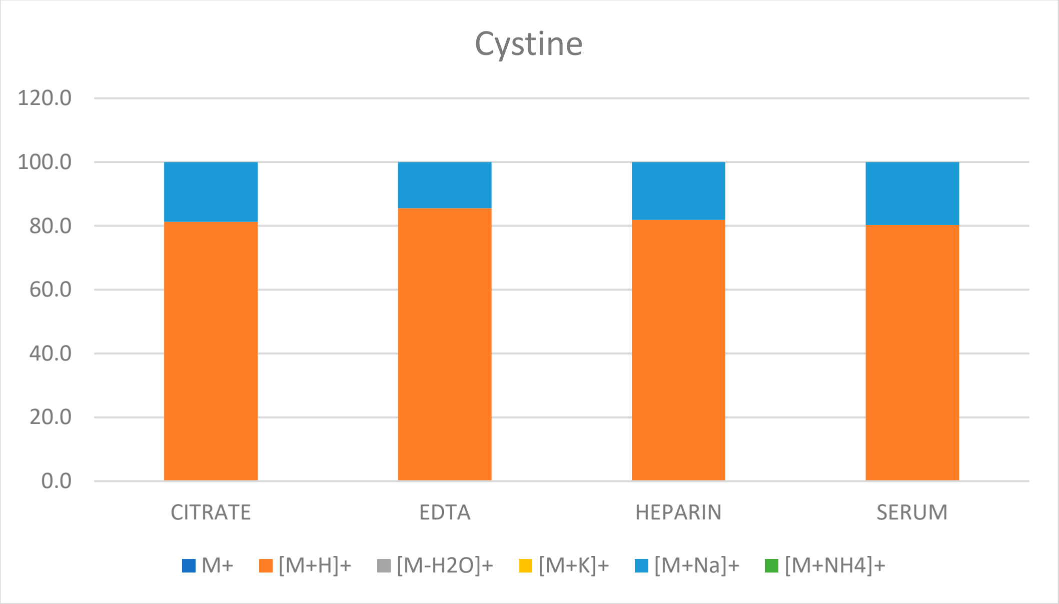
Cystine
120.0
100.0
80.0
60.0
40.0
20.0
0.0
CITRATE
EDTA
HEPARIN
SERUM
M+
[M+H]+
[M-H2O]+
[M+K]+
[M+Na]+
[M+NH4]+

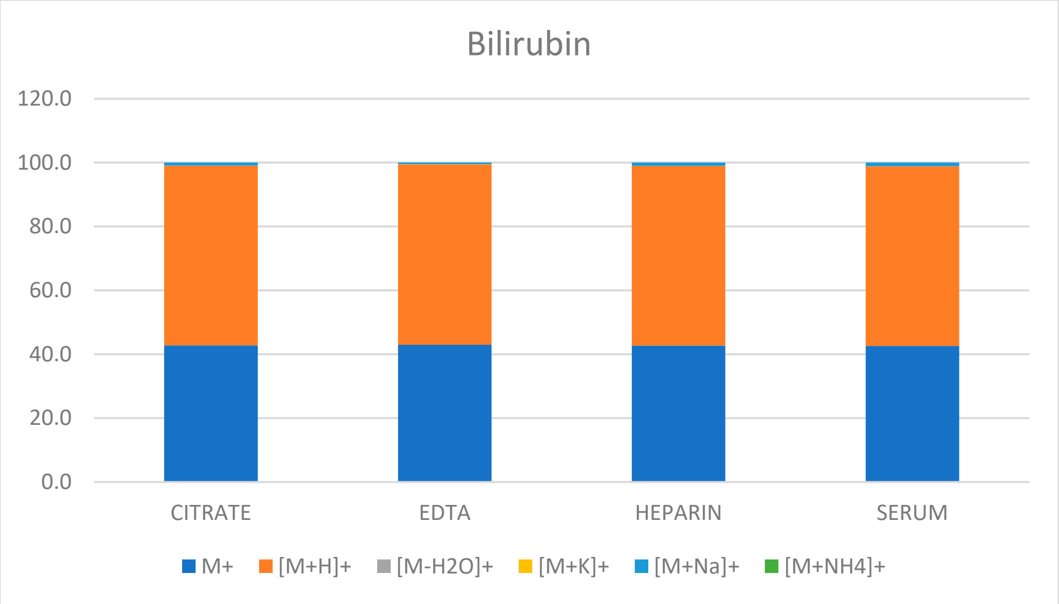
Bilirubin
120.0
100.0
80.0
60.0
40.0
20.0
0.0
CITRATE
EDTA
HEPARIN
SERUM
M+
[M+H]+
[M-H2O]+
[M+K]+
[M+Na]+
[M+NH4]+

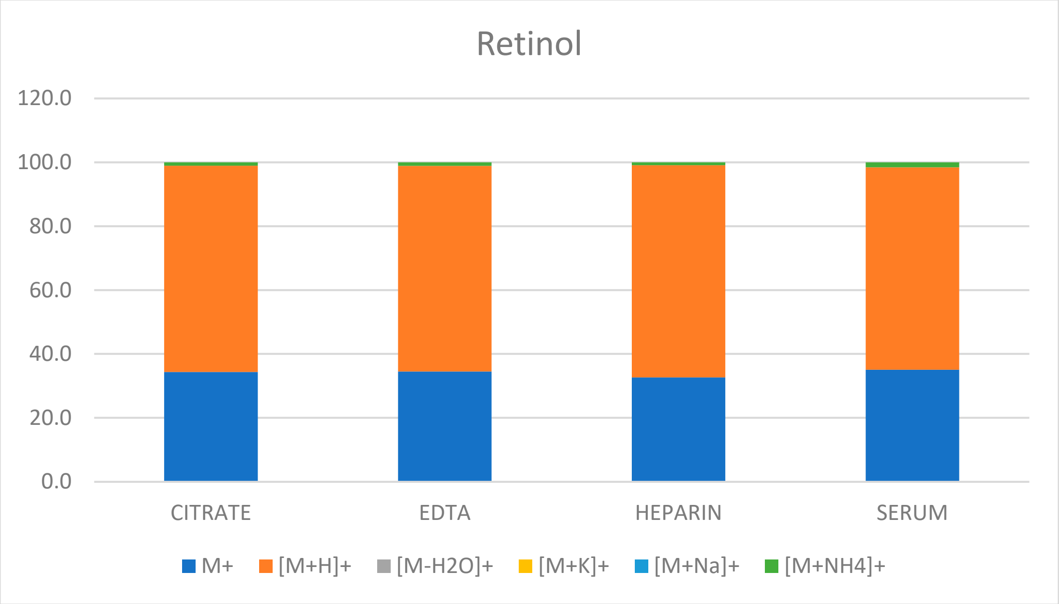
Retinol
120.0
100.0
80.0
60.0
40.0
20.0
0.0
CITRATE
EDTA
HEPARIN
SERUM
M+
[M+H]+
[M-H2O]+
[M+K]+
[M+Na]+
[M+NH4]+

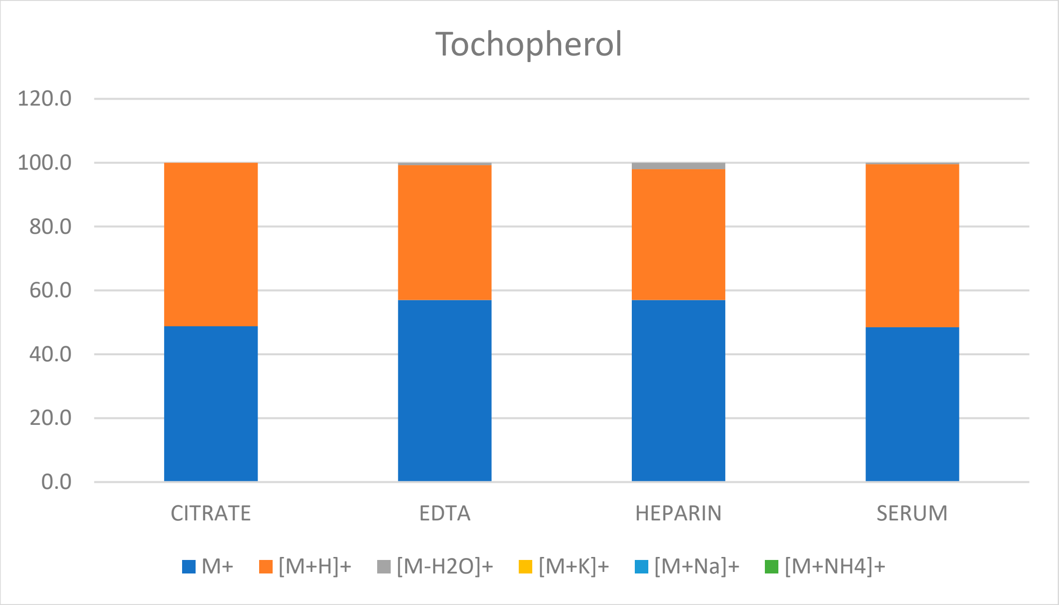
Tochopherol
120.0
100.0
80.0
60.0
40.0
20.0
0.0
CITRATE
EDTA
HEPARIN
SERUM
M+
[M+H]+
[M-H2O]+
[M+K]+
[M+Na]+
[M+NH4]+

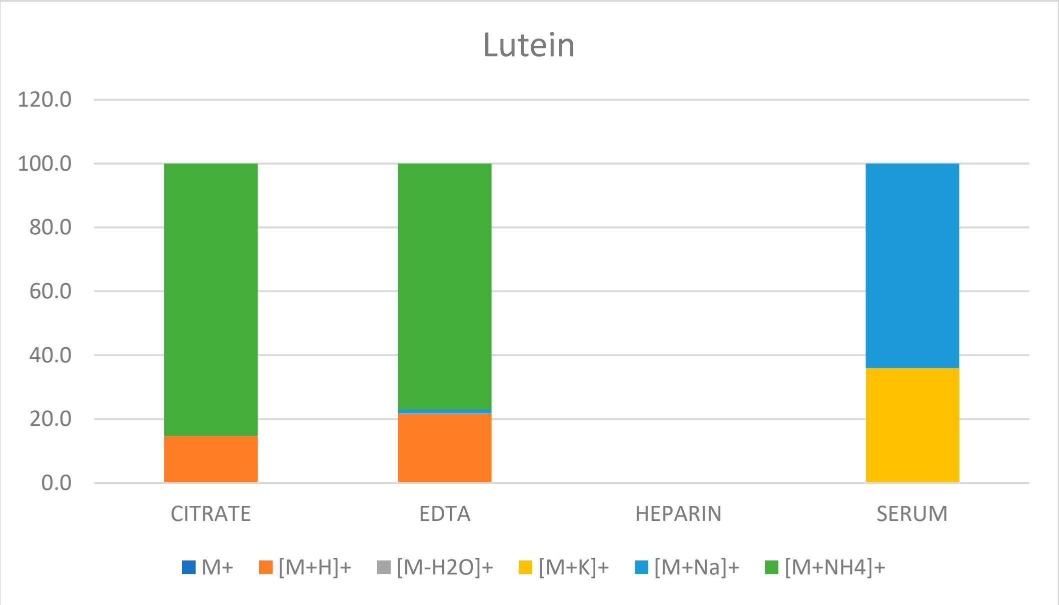
Lutein
120.0
100.0
80.0
60.0
40.0
20.0
0.0
CITRATE
EDTA
HEPARIN
SERUM
M+
[M+H]+
[M-H2O]+
[M+K]+
[M+Na]+
[M+NH4]+

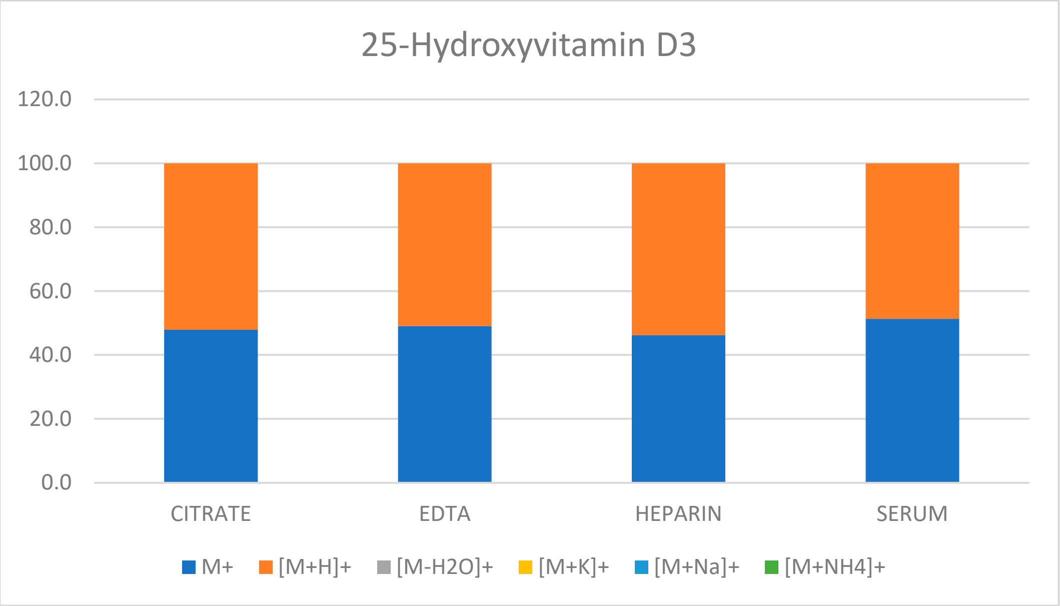
25-Hydroxyvitamin D3
120.0
100.0
80.0
60.0
40.0
20.0
0.0
CITRATE
EDTA
HEPARIN
SERUM
M+
[M+H]+
[M-H2O]+
[M+K]+
[M+Na]+
[M+NH4]+

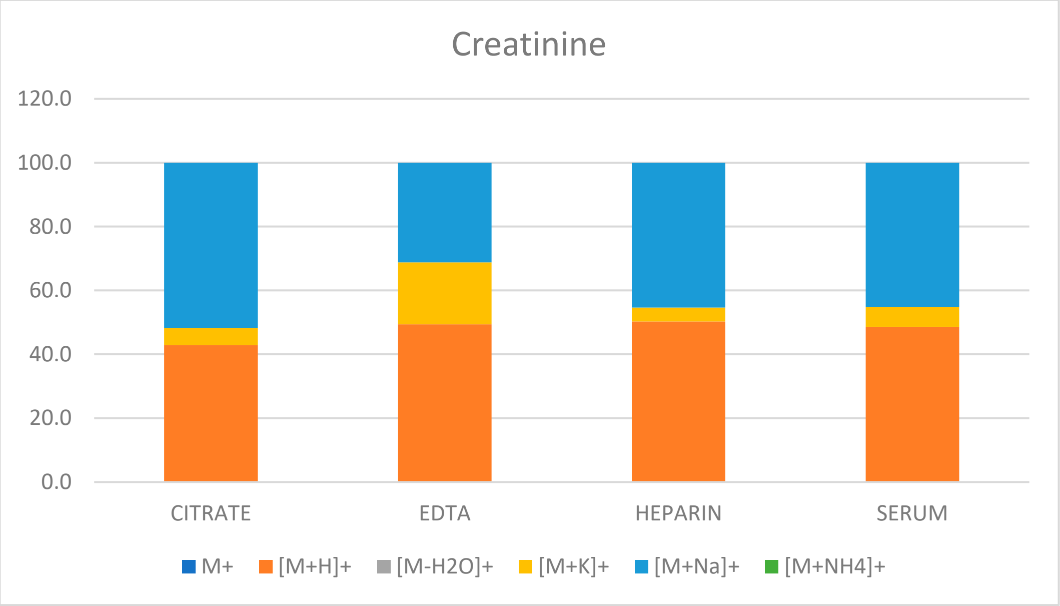
Creatinine
120.0
100.0
80.0
60.0
40.0
20.0
0.0
CITRATE
EDTA
HEPARIN
SERUM
M+
[M+H]+
[M-H2O]+
[M+K]+
[M+Na]+
[M+NH4]+

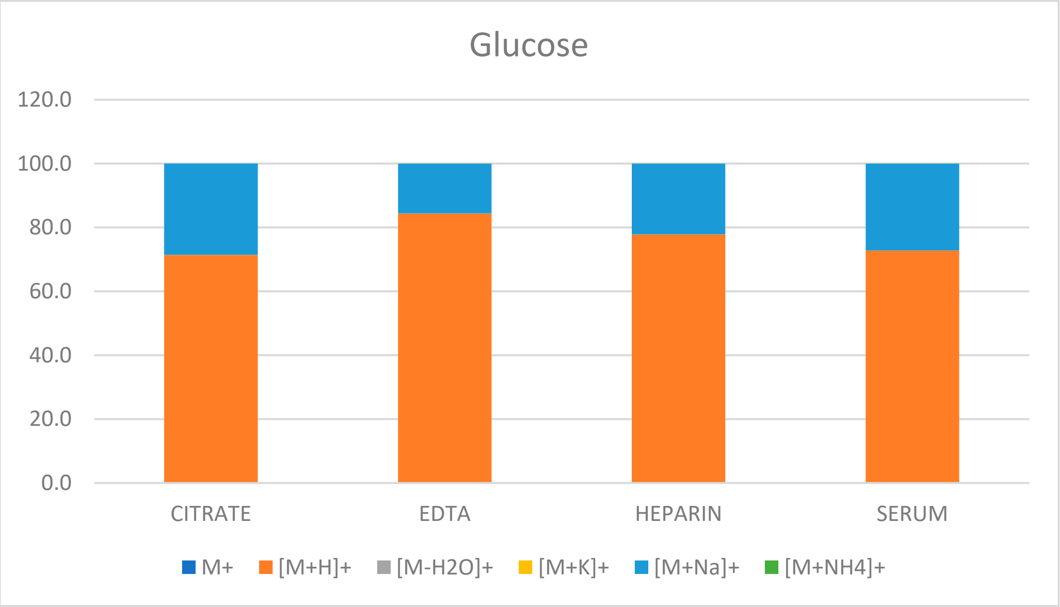
Glucose
120.0
100.0
80.0
60.0
40.0
20.0
0.0
CITRATE
EDTA
HEPARIN
SERUM
M+
[M+H]+
[M-H2O]+
[M+K]+
[M+Na]+
[M+NH4]+

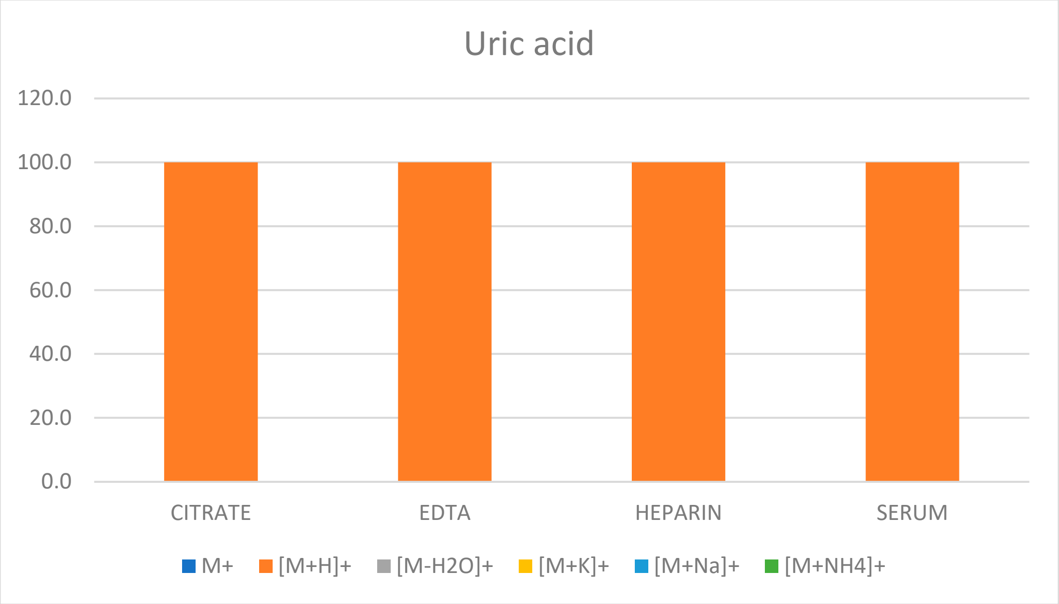
Uric acid
120.0
100.0
80.0
60.0
40.0
20.0
0.0
CITRATE
EDTA
HEPARIN
SERUM
M+
[M+H]+
[M-H2O]+
[M+K]+
[M+Na]+
[M+NH4]+

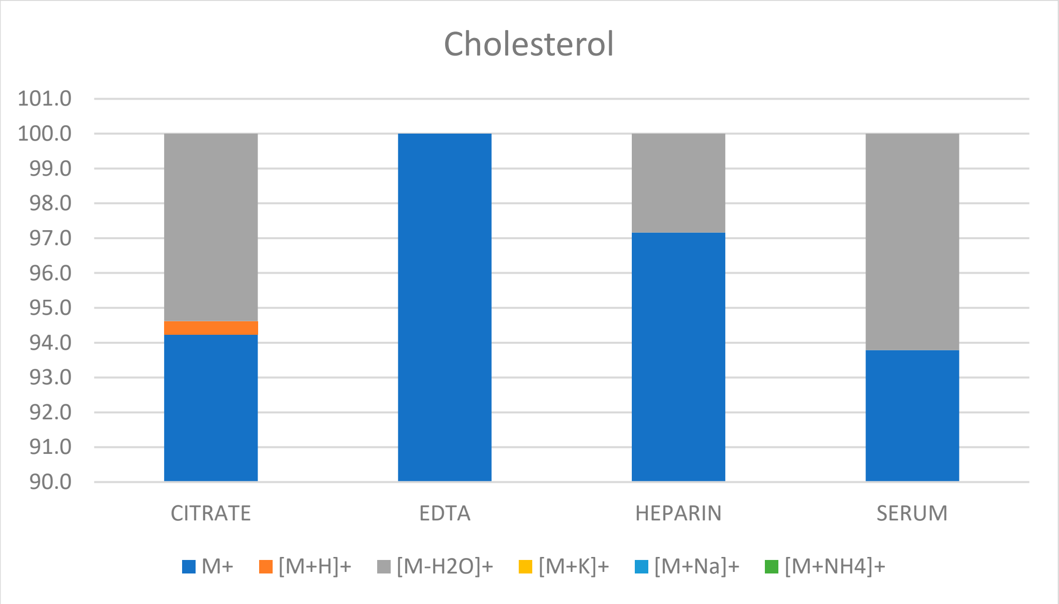
Cholesterol
101.0
100.0
99.0
98.0
97.0
96.0
95.0
94.0
93.0
92.0
91.0
90.0
CITRATE
EDTA
HEPARIN
SERUM
M+
[M+H]+
[M-H2O]+
[M+K]+
[M+Na]+
[M+NH4]+

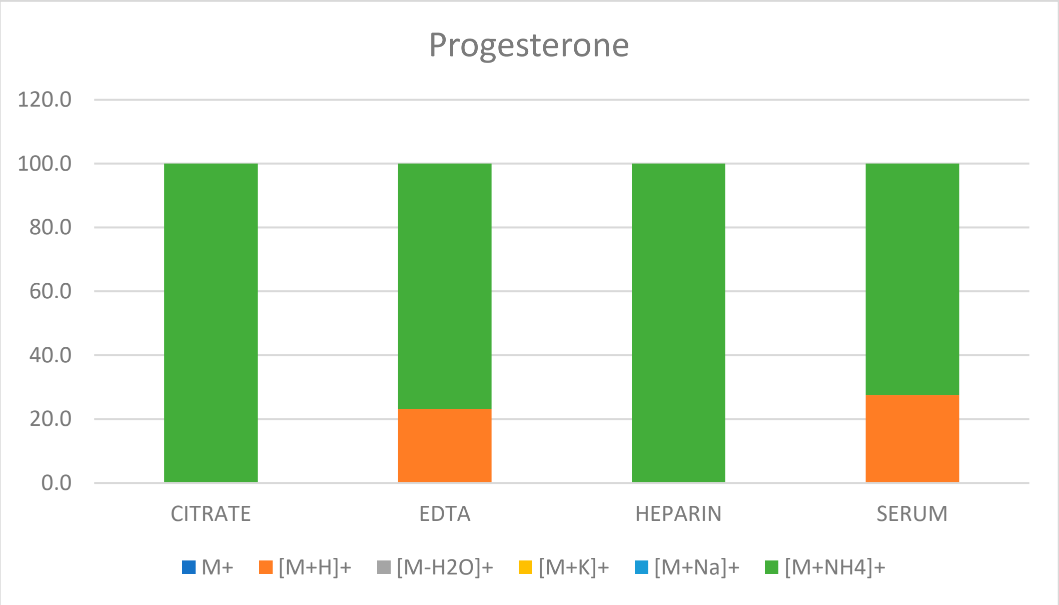
Progesterone
120.0
100.0
80.0
60.0
40.0
20.0
0.0
CITRATE
EDTA
HEPARIN
SERUM
M+
[M+H]+
[M-H2O]+
[M+K]+
[M+Na]+
[M+NH4]+

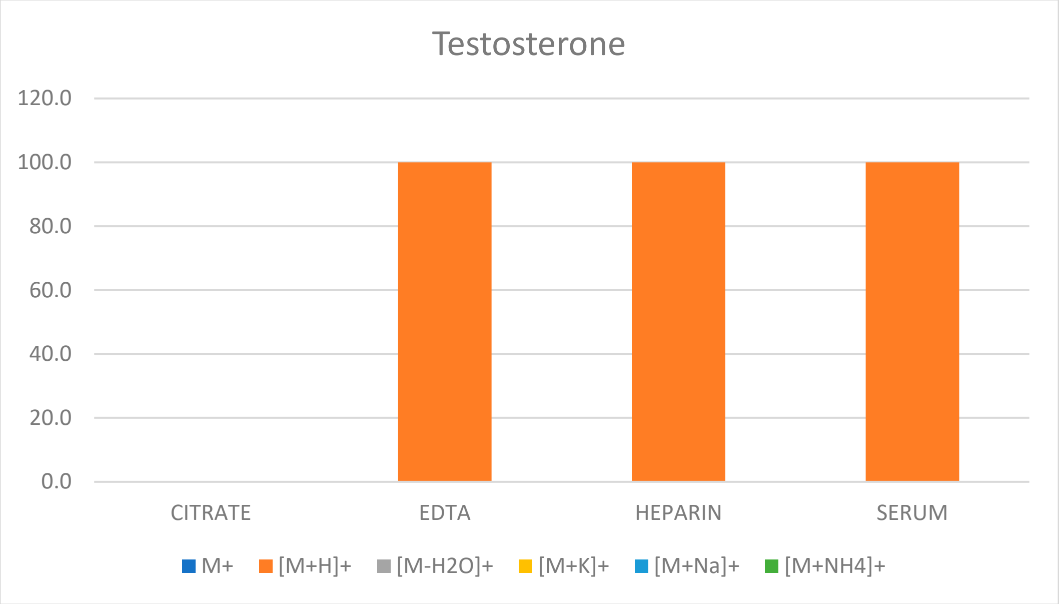
Testosterone
120.0
100.0
80.0
60.0
40.0
20.0
0.0
CITRATE
EDTA
HEPARIN
SERUM
M+
[M+H]+
[M-H2O]+
[M+K]+
[M+Na]+
[M+NH4]+

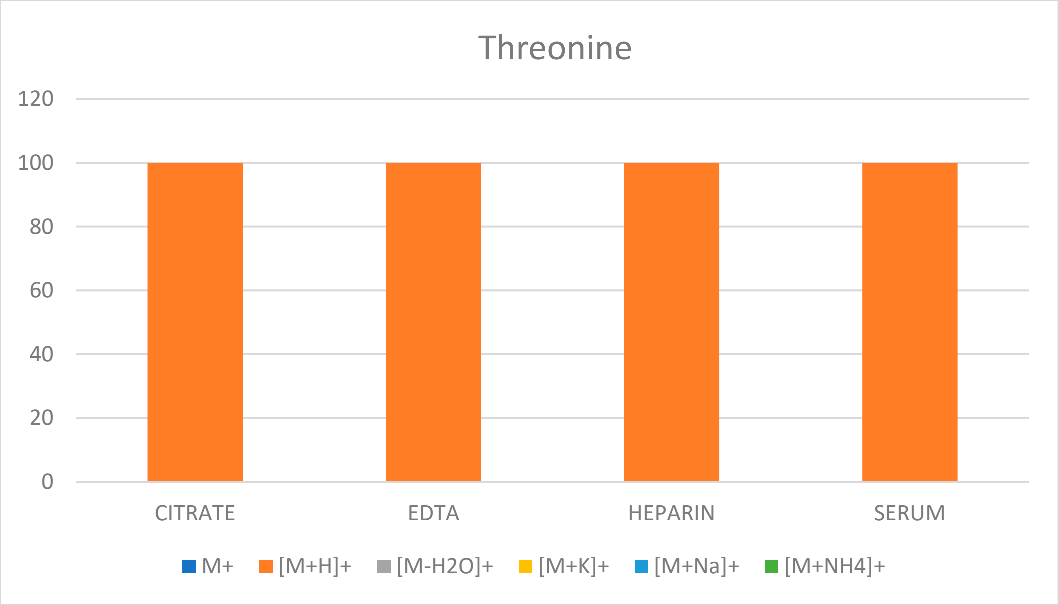
Threonine
120
100
80
60
40
20
0
CITRATE
EDTA
HEPARIN
SERUM
M+
[M+H]+
[M-H2O]+
[M+K]+
[M+Na]+
[M+NH4]+

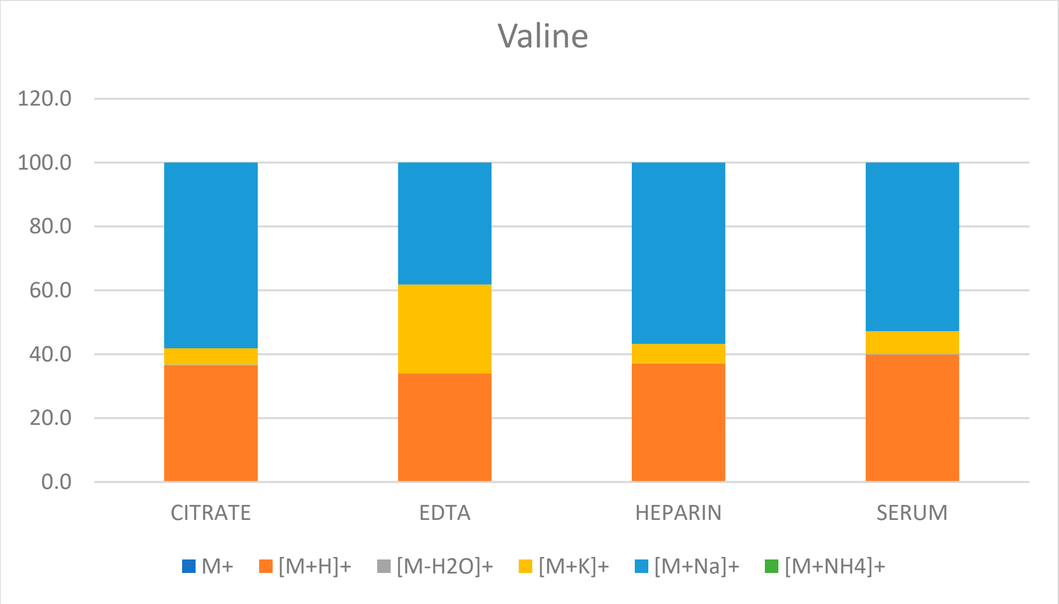
Valine
120.0
100.0
80.0
60.0
40.0
20.0
0.0
CITRATE
EDTA
HEPARIN
SERUM
M+
[M+H]+
[M-H2O]+
[M+K]+
[M+Na]+
[M+NH4]+
