# Supplementary material for: Investigating Variability in Metabolomics: A Comparative Study of Analytical Platforms and Blood Matrices Using HPLC-HRMS
Source: Molecules. 2026 Feb 28;31(5):814. doi: 10.3390/molecules31050814 (PMC12986168; doi:10.3390/molecules31050814)
Supplement: Supplementary file 1 [file molecules-31-00814-s001.zip › Workflow Compound Discoverer.pdf]

Investigating Variability in Metabolomics: A Comparative Study of Analytical Platforms and Blood Matrices Using LC-HRMS

Giulia Guerra [1], Alessio Polymeropoulos [2], Elisabetta Venturelli [3,*], Veronica Huber [4], Francesco Segrado[3], Daniele Morelli [5] and Sabina Sieri [1]

1 Epidemiology and Prevention Unit, Fondazione IRCCS Istituto Nazionale dei Tumori di Milano, 20133 Milan, Italy; giulia.guerra@istitutotumori.mi.it (G.G.) https://orcid.org/0009-0003-4546-9718; sabina.sieri@istitutotumori.mi.it (S.S.) https://orcid.org/0000-0001-5201-172X
2 Biostatistics for Clinical Research, Fondazione IRCCS Istituto Nazionale dei Tumori di Milano, 20133 Milan, Italy; alessio.polymeropoulos@istitutotumori.mi.it https://orcid.org/0000-0002-2652-3964
3 Nutrition Research and Metabolomics Unit, Fondazione IRCCS Istituto Nazionale dei Tumori di Milano, 20133 Milan, Italy; francesco.segrado@istitutotumori.mi.it https://orcid.org/0000-0002-1270-4609
4 Unit of Translational Immunology, Fondazione IRCCS Istituto Nazionale dei Tumori di Milano, 20133 Milan, Italy; veronica.huber@istitutotumori.mi.it https://orcid.org/0000-0001-6304-3575
5 Laboratory Medicine Department, Fondazione IRCCS Istituto Nazionale dei Tumori di Milano, 20133 Milan, Italy; daniele.morelli@istitutotumori.mi.it https://orcid.org/0000-0002-1823-3764
* Correspondence: elisabetta.venturelli@istitutotumori.mi.it; Tel.: +39-02-2390-3745 https://orcid.org/0000-0002-7427-7032

Search name: PhD_matrix_C18+
Search description: Untargeted Metabolomics workflow: Find and identify the differences between samples.
- Performs retention time alignment, unknown compound detection, and compound grouping across all samples. Predicts elemental compositions for all compounds, fills gaps across all samples, and hides chemical background (using Blank samples). Identifies compounds using mzCloud (ddMS2) and ChemSpider (formula or exact mass). Also performs similarity search for all compounds with ddMS2 data using mzCloud. Applies mzLogic algorithm to rank order ChemSpider results. Maps compounds to biological pathways using Metabolika. Applies QC-based batch normalization if QC samples are available. Calculates differential analysis (t-test or ANOVA), determines p-values, adjusted p-values, ratios, fold change, CV, etc.).
Search date: 19/01/2024 17:01:51
Created with Discoverer version: 3.3.1.111

```
[Input Files (6)]
    -->Select Spectra (33)

    [Select Spectra (33)]
        -->Align Retention Times (ChromAlign) (51)

        [Align Retention Times (ChromAlign) (51)]
            -->Create Mass Trace (39)
            -->Detect Compounds (50)

            [Detect Compounds (50)]
                -->Group Compounds (31)

                [Group Compounds (31)]
                    -->Fill Gaps (32)
                    -->Search Neutral Losses (52)
                    -->Assign Compound Annotations (25)
```

-->Search mzCloud (47)
-->Search mzVault (49)
-->Map to Metabolika Pathways (34)
-->Search Mass Lists (40)
-->Search ChemSpider (48)
-->Predict Compositions (29)

[Fill Gaps (32)]
-->Apply SERRF QC Correction (55)

[Apply SERRF QC Correction (55)]
-->Mark Background Compounds (28)

[Assign Compound Annotations (25)]
-->Generate Molecular Networks (42)

[Map to Metabolika Pathways (34)]
-->Apply Spectral Distance (41)
-->Apply mzLogic (54)

[Search Mass Lists (40)]
-->Apply Spectral Distance (41)
-->Apply mzLogic (54)

[Search ChemSpider (48)]
-->Apply Spectral Distance (41)
-->Apply mzLogic (54)

[Create Mass Trace (39)]

[Mark Background Compounds (28)]

[Search Neutral Losses (52)]

[Generate Molecular Networks (42)]

[Search mzCloud (47)]

[Search mzVault (49)]

[Apply Spectral Distance (41)]

[Apply mzLogic (54)]

[Predict Compositions (29)]

[Differential Analysis (17)]

[Descriptive Statistics (43)]

Processing node 6: Input Files

Processing node 33: Select Spectra
----
1. Spectrum Properties Filter:
- Lower RT Limit: 0.2
- Upper RT Limit: 43
- First Scan: 0
- Last Scan: 0
- Ignore Specified Scans: (not specified)
- Lowest Charge State: 0
- Highest Charge State: 0
- Min. Precursor Mass: 0 Da
- Max. Precursor Mass: 5000 Da
- Total Intensity Threshold: 0
- Minimum Peak Count: 1

2. Scan Event Filters:
- Mass Analyzer: (not specified)
- MS Order: Any
- Activation Type: (not specified)
- Min. Collision Energy: 0
- Max. Collision Energy: 1000
- Scan Type: Any
- Polarity Mode: Any
- MS1 Mass Range: (not specified)
- FAIMS CV: (not specified)

3. Peak Filters:
- S/N Threshold (FT-only): 1.5

4. Replacements for Unrecognized Properties:
- Unrecognized Charge Replacements: 1
- Unrecognized Mass Analyzer Replacements: ITMS
- Unrecognized MS Order Replacements: MS2
- Unrecognized Activation Type Replacements: CID
- Unrecognized Polarity Replacements: +
- Unrecognized MS Resolution@200 Replacements: 60000
- Unrecognized MSn Resolution@200 Replacements: 30000

5. General Settings:
- Precursor Selection: Use MS(n - 1) Precursor
- Use Isotope Pattern in Precursor Reevaluation: True
- Provide Profile Spectra: Automatic
- Store Chromatograms: False

Processing node 51: Align Retention Times (ChromAlign)
----
1. General Settings:
- Reference File: (not specified)

Processing node 39: Create Mass Trace
----
1. General Settings:
- Trace Type: BPC
- MS Order: MS1
- Polarity: +
- Custom Label: BP+

2. XIC Settings:
- Mass [Da]: 0
- Mass Tolerance: 10 ppm

Processing node 50: Detect Compounds
----
1. General Settings:
- Mass Tolerance [ppm]: 7 ppm
- Min. Peak Intensity: 30000
- Min. # Scans per Peak: 5
- Use Most Intense Isotope Only: True

2. Trace Detection:
- Max. Number of Gaps to Correct: 2
- Min. Number of Adjacent Non-Zeros: 2

3. Peak Detection:
- Chromatographic S/N Threshold: 1.5
- Remove Baseline: True
- Gap Ratio Threshold: 0.35
- Max. Peak Width [min]: 1
- Min. Relative Valley Depth: 0.1

4. Isotope Pattern Detection:
- Group Isotopes for: Br; Cl
- Use Peak Quality for Isotope Grouping: True
- Filter out Features with Bad Peaks Only: True
- Zig-Zag Index Threshold: 0.2
- Jaggedness Threshold: 0.4
- Modality Threshold: 0.9
- Remove Potentially False Positive Isotopes: False

5. Compound Detection:
- Ions: [M+H]+1; [M+K]+1; [M+Na]+1; [M+NH4]+1
- Base Ions: [M+H]+1; [M+NH4]+1; [M-H]-1
- Remove Singlets: False

6. AcquireX Settings:
- Detect Persistent Background Ions: False

Processing node 31: Group Compounds
----
1. General Settings:
- Mass Tolerance: 7 ppm
- RT Tolerance [min]: 0.2
- Align Peaks: False
- Preferred Ions: [M+H]+1; [M+K]+1; [M+Na]+1; [M+NH4]+1
- Area Integration: All Ions

2. Peak Rating Contributions:
- Area Contribution: 3
- CV Contribution: 10
- FWHM to Base Contribution: 5
- Jaggedness Contribution: 5
- Modality Contribution: 5
- Zig-Zag Index Contribution: 5

3. Peak Rating Filter:
- Peak Rating Threshold: 4
- Number of Files: 1

Processing node 32: Fill Gaps
----
1. General Settings:
- Mass Tolerance: 7 ppm
- S/N Threshold: 1.5
- Use Real Peak Detection: True
- Apply Restrictive Gap Filling: True

Processing node 55: Apply SERRF QC Correction
----
1. General Settings:
- Min. QC Coverage [%]: 60
- Max. QC Area RSD [%]: 35
- Max. Corrected QC Area RSD [%]: 30
- Max. # Files Between QC Files: 10
- Correct Blank Files: False
- # Batches: 1
- Interpolate Gap-filled QC Areas: True

2. Random Forest Settings:
- # Trees: 200

Processing node 28: Mark Background Compounds
----
1. General Settings:
- Max. Sample/Blank: 5
- Max. Blank/Sample: 0
- Hide Background: True

Processing node 52: Search Neutral Losses
----
1. General Settings:
- Neutral Losses:
    Br (Br, 78.92)
    C2H3 (C2 H3, 27.02)
    C2H3O2 (C2 H3 O2, 59.01)
    C2H4N (C2 H4 N, 42.03)
    C2H5 (C2 H5, 29.04)
    C2H5O (C2 H5 O, 45.03)
    C2H5OH (C2 H6 O, 46.04)
    C2H6 (C2 H6, 30.05)
    C3H7 (C3 H7, 43.05)
    C4H10 (C4 H10, 58.08)
    C4H7 (C4 H7, 55.05)
    C4H8 (C4 H8, 56.06)
    C4H9 (C4 H9, 57.07)
    CH2 (C H2, 14.02)
    CH2C=O (C2 H2 O, 42.01)
    CH3 (C H3, 15.02)
    CH3CH=CH2 (C3 H6, 42.05)
    CH3CO (C2 H3 O, 43.02)
    CH3COOH (C2 H4 O2, 60.02)
    CH3O (C H3 O, 31.02)
    CH3OH (C H4 O, 32.03)
    CH4 (C H4, 16.03)
    CH5O (C H5 O, 33.03)
    Cl (Cl, 34.97)
    CO (C O, 27.99)
    CO2 (C O2, 43.99)
    CO3 (C O3, 59.98)
    CONH2 (C H2 N O, 44.01)
    Deoxyhexose (C6 H10 O4, 146.06)
    F (F, 19.00)
    Fatty_Acid_16:0 (C16 H32 O2, 256.24)
    Fatty_Acid_16:1 (C16 H30 O2, 254.22)
    Fatty_Acid_18:0 (C18 H36 O2, 284.27)
    Fatty_Acid_18:1 (C18 H34 O2, 282.26)
    Fatty_Acid_18:2 (C18 H32 O2, 280.24)
    Fatty_Acid_18:3 (C18 H30 O2, 278.22)
    Fatty_Acid_20:4 (C20 H32 O2, 304.24)
    Fatty_Acid_22:5 (C22 H34 O2, 330.26)
    Fatty_Acid_22:6 (C22 H32 O2, 328.24)
    Glucuronic Acid (C6 H10 O7, 194.04)
    Glucuronide (C6 H8 O6, 176.03)
    H (H, 1.01)
    H2 (H2, 2.02)
    H2C=CH2 (C2 H4, 28.03)
    H2C=O (C H2 O, 30.01)
    H2O (H2 O, 18.01)

H2S (H2 S, 33.99)
HBr (H Br, 79.93)
HCCH (C2 H2, 26.02)
HCl (H Cl, 35.98)
HCN (C H N, 27.01)
Hexose (C6 H10 O5, 162.05)
Hexose-Hexose (C12 H20 O10, 324.11)
HF (H F, 20.01)
HI (H I, 127.91)
HS (H S, 32.98)
I (I, 126.90)
NH3 (H3 N, 17.03)
NO (N O, 30.00)
NO2 (N O2, 45.99)
OH (H O, 17.00)
Pentose (C5 H8 O4, 132.04)
Pentose-Hexose (C11 H18 O9, 294.10)
Pentose-Pentose (C10 H16 O8, 264.08)
Pyroglutamic Acid (C5 H7 N O3, 129.04)
S (S, 31.97)
SO (O S, 47.97)
SO2 (O2 S, 63.96)
y-GluAlaGly-2H (C10 H15 N3 O6, 273.10)

- High Acc. Mass Tolerance: 2.5 mmu
- Low Acc. Mass Tolerance: 0.5 Da
- S/N Threshold: 3
- Use DIA Scans for Search: False

Processing node 25: Assign Compound Annotations
----
1. General Settings:
- Mass Tolerance: 7 ppm

2. Data Sources:
- Data Source #1: mzVault Search
- Data Source #2: mzCloud Search
- Data Source #3: MassList Search
- Data Source #4: Predicted Compositions
- Data Source #5: Metabolika Search
- Data Source #6: ChemSpider Search
- Data Source #7: (not specified)

3. Scoring Rules:
- Use mzLogic: False
- Use Spectral Distance: True
- SFit Threshold: 20
- SFit Range: 20

4. Reprocessing:
- Clear Names: False

Processing node 42: Generate Molecular Networks
----
1. Spectral Similarity:
- Use Full MSn Tree: True
- Match Mass Shift: True
- Match Transformations: True
- Variate Transformations: False
- S/N Threshold: 3
- Mass Tolerance: 7 ppm
- Min. Fragment m/z: 50

2. Transformations:
- Phase I:
    Dehydration (H2 O ->)
    Desaturation (H2 ->)
    Hydration (-> H2 O)
    Oxidation (-> O)
    Reduction (-> H2)
- Phase II:
    Acetylation (H -> C2 H3 O)
    Glucoside Conjugation (H -> C6 H11 O5)
    Glucuronide Conjugation (H -> C6 H9 O6)
    Methylation (H -> C H3)
    Ornitine Conjugation (H O -> C5 H11 N2 O2)
    Palmitoyl Conjugation (H -> C16 H31 O)
    Sulfation (H -> H O3 S)
- Others: (not specified)
- Max. # Phase II: 1
- Max. # All Steps: 3

3. Applied View Filters:
- Require Transformation: True
- Require MSn: True
- Min. MSn Score: 50
- Min. MSn Coverage: 50
- Min. Fragments: 2

4. Applied Thresholds:
- Require Transformation: False
- Require MSn: False
- Min. MSn Score: 20
- Min. MSn Coverage: 20
- Min. Fragments: 0

Processing node 47: Search mzCloud
----
1. General Settings:
- Compound Classes: All
- Precursor Mass Tolerance: 7 ppm
- FT Fragment Mass Tolerance: 15 ppm

- IT Fragment Mass Tolerance: 0.4 Da
- Library: Autoprocessed; Reference
- Post Processing: Recalibrated
- Max. # Results: 15
- Annotate Matching Fragments: True
- Search MSn Tree: False

2. DDA Search:
- Identity Search: Cosine
- Match Activation Type: True
- Match Activation Energy: Match with Tolerance
- Activation Energy Tolerance: 30
- Apply Intensity Threshold: True
- Similarity Search: Similarity Forward
- Match Factor Threshold: 40

3. DIA Search:
- Use DIA Scans for Search: False
- Max. Isolation Width [Da]: 500
- Match Activation Type: False
- Match Activation Energy: Any
- Activation Energy Tolerance: 100
- Apply Intensity Threshold: False
- Match Factor Threshold: 20

Processing node 49: Search mzVault
----
1. Search Settings:
- mzVault Library: Bamba lab 34 lipid mediators library stepped NCE 10 30 45.db|Bamba lab 598 polar metabolites stepped NCE 10 30 45.db|Custom mzVault Library.db|LipidBlast-VS68-Pos.db|mzCloud Offline for mzVault_Endogenous_2021B.db|mzCloud_Offline for mzVault_Autoprocessed_2021B.db|mzCloud_Offline for mzVault_Endogenous-Autoprocessed_2021B.db|mzCloud_Offline for mzVault_Reference_2021B.db
- Max. # Results: 10
- Match Factor Threshold: 30
- Search Algorithm: HighChem HighRes
- Match Analyzer Type: False
- IT Fragment Mass Tolerance: 0.4 Da
- FT Fragment Mass Tolerance: 15 ppm
- Use Retention Time: False
- Precursor Mass Tolerance: 7 ppm
- Apply Intensity Threshold: False
- Match Ionization Method: False
- Ion Activation Energy Tolerance: 30
- Match Ion Activation Energy: Any
- Match Ion Activation Type: True
- Compound Classes: All
- Remove Precursor Ion: False
- RT Tolerance [min]: 0.4

Processing node 34: Map to Metabolika Pathways
----
1. Search Settings:
- Metabolika Pathways: (3R)-linalool biosynthesis.metabolika|2-nitrobenzoate degradation I.metabolika|2-oxobutanoate degradation I.metabolika|3-phenylpropanoate and 3-(3-hydroxyphenyl)propanoate degradation.metabolika|3-phenylpropanoate degradation.metabolika|Acetyl-CoA fermentation to butanoate II.metabolika|Adenosylcobalamin biosynthesis I (anaerobic).metabolika|Adenosylcobalamin biosynthesis II (aerobic).metabolika|Allantoin degradation IV (anaerobic).metabolika|Allantoin degradation to glyoxylate I.metabolika|Allantoin degradation to glyoxylate II.metabolika|Allantoin degradation to glyoxylate III.metabolika|Ammonia assimilation cycle I.metabolika|Ammonia assimilation cycle III.metabolika|Ammonia oxidation IV (autotrophic ammonia oxidizers).metabolika|Anaerobic aromatic compound degradation (Thauera aromatica).metabolika|Anaerobic energy metabolism (invertebrates, mitochondrial).metabolika|Arachidonate biosynthesis III (6-desaturase, mammals).metabolika|Archaetidylinositol biosynthesis.metabolika|Archaetidylserine and archaetidylethanolamine biosynthesis.metabolika|Arginine, ornithine and proline interconversion.metabolika|Aromatic compounds degradation via ss-ketoadipate.metabolika|Aspartate superpathway.metabolika|B-carotene biosynthesis (engineered).metabolika|Bacillibactin biosynthesis.metabolika|Benzoate biosynthesis I (CoA-dependent, ss-oxidative).metabolika|Benzoate biosynthesis III (CoA-dependent, non-ss-oxidative).metabolika|Benzoate fermentation (to acetate and cyclohexane carboxylate).metabolika|Biotin biosynthesis I.metabolika|Biotin biosynthesis II.metabolika|Bitter acids biosynthesis.metabolika|Caffeine degradation IV (bacteria, via demethylation and oxidation).metabolika|Cardiolipin and phosphatidylethanolamine biosynthesis (Xanthomonas).metabolika|Catechol degradation I (meta-cleavage pathway).metabolika|Catechol degradation II (meta-cleavage pathway).metabolika|Catechol degradation III (ortho-cleavage pathway).metabolika|Cellulose and hemicellulose degradation (cellulolosome).metabolika|Chitin biosynthesis.metabolika|Cholesterol biosynthesis I.metabolika|Cholesterol biosynthesis II (via 24,25-dihydrolanosterol).metabolika|Cholesterol biosynthesis III (via desmosterol).metabolika|Choline degradation IV.metabolika|Choline-O-sulfate degradation.metabolika|Chondroitin sulfate biosynthesis.metabolika|Chorismate biosynthesis I.metabolika|Chorismate biosynthesis II (archaea).metabolika|Colanic acid building blocks biosynthesis.metabolika|Crotonate fermentation (to acetate and cyclohexane carboxylate).metabolika|Curcuminoid biosynthesis.metabolika|D-serine metabolism.metabolika|Dermatan sulfate biosynthesis.metabolika|Enterobacterial common antigen biosynthesis.metabolika|Enterobactin biosynthesis.metabolika|G-butyrobetaine degradation.metabolika|GABA shunt.metabolika|Gamma-glutamyl cycle.metabolika|Gluconeogenesis II (Methanobacterium thermoautotrophicum).metabolika|Glycerol and glycerophosphodiester degradation.metabolika|Glycerol degradation to butanol.metabolika|Glycine biosynthesis II.metabolika|Heparan sulfate biosynthesis.metabolika|Hexitol fermentation to lactate, formate, ethanol and acetate.metabolika|Homolactic fermentation.metabolika|Hyperxanthone E biosynthesis.metabolika|Icosapentaenoate biosynthesis III (fungi).metabolika|Icosapentaenoate biosynthesis IV (bacteria).metabolika|Isoprene biosynthesis I.metabolika|Kanamycin biosynthesis.metabolika|Kauralexin biosynthesis.metabolika|Kdo transfer to lipid IVA III (Chlamydia).metabolika|Ketogluconate metabolism.metabolika|L-alanine fermentation to propanoate and acetate.metabolika|L-arginine biosynthesis I (via L-ornithine).metabolika|L-arginine degradation V (arginine deiminase pathway).metabolika|L-ascorbate biosynthesis V.metabolika|L-cysteine biosynthesis IV (from L-methionine).metabolika|L-cysteine biosynthesis IV (fungi).metabolika|L-glutamate and L-glutamine biosynthesis.metabolika|L-glutamate degradation IX (via 4-aminobutanoate).metabolika|L-glutamate degradation VII (to butanoate).metabolika|L-glutamate degradation VIII (to propanoate).metabolika|L-homoserine and L-methionine

biosynthesis.metabolika|L-methionine biosynthesis III.metabolika|L-methionine salvage cycle I (bacteria and plants).metabolika|L-methionine salvage cycle II (plants).metabolika|L-methionine salvage cycle III.metabolika|L-tryptophan degradation III (eukaryotic).metabolika|L-tryptophan degradation IX.metabolika|L-tryptophan degradation XI (mammalian, via kynurenine).metabolika|L-tryptophan degradation XII (Geobacillus).metabolika|L-tyrosine degradation IV (to 4-methylphenol).metabolika|Mandelate degradation to acetyl-CoA.metabolika|Meta cleavage pathway of aromatic compounds.metabolika|Methanobacterium thermoautotrophicum biosynthetic metabolism.metabolika|Methanol and methylamine oxidation to formaldehyde.metabolika|Methanol oxidation to carbon dioxide.metabolika|Methylglyoxal degradation IV.metabolika|MRNA capping II.metabolika|Myo-, chiro- and scillo-inositol degradation.metabolika|N-acetylglucosamine degradation II.metabolika|NAD biosynthesis II (from tryptophan).metabolika|NAD salvage pathway III.metabolika|Naphthalene degradation to acetyl-CoA.metabolika|Nitrifier denitrification.metabolika|Novobiocin biosynthesis.metabolika|O-antigen building blocks biosynthesis (E. coli).metabolika|Oxygenic photosynthesis.metabolika|P-cumate degradation.metabolika|P-cymene degradation.metabolika|Pentose phosphate pathway.metabolika|Peptidoglycan biosynthesis I (meso-diaminopimelate containing).metabolika|Peptidoglycan biosynthesis II (staphylococci).metabolika|Peptidoglycan biosynthesis III (mycobacteria).metabolika|Peptidoglycan biosynthesis IV (Enterococcus faecium).metabolika|Peptidoglycan biosynthesis V (ss-lactam resistance).metabolika|Phosphatidylglycerol biosynthesis I (plastidic).metabolika|Phosphatidylglycerol biosynthesis II (non-plastidic).metabolika|Plant sterol biosynthesis.metabolika|Polyisoprenoid biosynthesis (E. coli).metabolika|Purine nucleotides degradation I (plants).metabolika|Purine nucleotides degradation II (aerobic).metabolika|Pyrimidine nucleobases salvage II.metabolika|Pyruvate fermentation to acetate and alanine.metabolika|Pyruvate fermentation to acetate and lactate I.metabolika|Pyruvate fermentation to acetate and lactate II.metabolika|Pyruvate fermentation to acetate I.metabolika|Pyruvate fermentation to acetate III.metabolika|Pyruvate fermentation to acetate IV.metabolika|Pyruvate fermentation to acetate V.metabolika|Pyruvate fermentation to acetate VI.metabolika|Pyruvate fermentation to acetate VII.metabolika|Reactive oxygen species degradation.metabolika|S-adenosyl-L-methionine cycle I.metabolika|Salicylate glucosides biosynthesis I.metabolika|Sphingolipid biosynthesis (mammals).metabolika|Sucrose biosynthesis I (from photosynthesis).metabolika|Sulfate reduction I (assimilatory).metabolika|Superpathway avenacin A biosynthesis.metabolika|Superpathway NADNADP - NADHNADPH interconversion (yeast).metabolika|Superpathway of (Kdo)2-lipid A biosynthesis.metabolika|Superpathway of (R,R)-butanediol biosynthesis.metabolika|Superpathway of 1D-myo-inositol hexakisphosphate biosynthesis (plants).metabolika|Superpathway of 2,3-butanediol biosynthesis.metabolika|Superpathway of 4-aminobutanoate degradation.metabolika|Superpathway of 4-hydroxybenzoate biosynthesis (yeast).metabolika|Superpathway of 5-aminoimidazole ribonucleotide biosynthesis.metabolika|Superpathway of acetate utilization and formation.metabolika|Superpathway of acetyl-CoA biosynthesis.metabolika|Superpathway of acrylonitrile degradation.metabolika|Superpathway of adenosine nucleotides de novo biosynthesis I.metabolika|Superpathway of adenosine nucleotides de novo biosynthesis II.metabolika|Superpathway of aerobic toluene degradation.metabolika|Superpathway of aflatoxin biosynthesis.metabolika|Superpathway of allantoin degradation in plants.metabolika|Superpathway of allantoin degradation in yeast.metabolika|Superpathway of Allium flavor precursors.metabolika|Superpathway of ammonia assimilation (plants).metabolika|Superpathway of anaerobic energy metabolism (invertebrates).metabolika|Superpathway of anaerobic sucrose degradation.metabolika|Superpathway of anthocyanin biosynthesis (from cyanidin and cyanidin 3-O-glucoside).metabolika|Superpathway of anthocyanin biosynthesis (from delphinidin 3-O-glucoside).metabolika|Superpathway of anthocyanin biosynthesis (from pelargonidin 3-O-glucoside).metabolika|Superpathway of arginine and polyamine biosynthesis.metabolika|Superpathway of aromatic amino acid

biosynthesis.metabolika|Superpathway of aromatic compound degradation via 2-oxopent-4-enoate.metabolika|Superpathway of aromatic compound degradation via 3-oxoadipate.metabolika|Superpathway of atrazine degradation.metabolika|Superpathway of bacteriochlorophyll a biosynthesis.metabolika|Superpathway of benzoxazinoid glucosides biosynthesis.metabolika|Superpathway of betalain biosynthesis.metabolika|Superpathway of branched chain amino acid biosynthesis.metabolika|Superpathway of butirocin biosynthesis.metabolika|Superpathway of C1 compounds oxidation to CO2.metabolika|Superpathway of C28 brassinosteroid biosynthesis.metabolika|Superpathway of candicidin biosynthesis.metabolika|Superpathway of carotenoid biosynthesis.metabolika|Superpathway of CDP-glucose-derived O-antigen building blocks biosynthesis.metabolika|Superpathway of cholesterol biosynthesis.metabolika|Superpathway of cholesterol degradation I (cholesterol oxidase).metabolika|Superpathway of cholesterol degradation II (cholesterol dehydrogenase).metabolika|Superpathway of choline biosynthesis.metabolika|Superpathway of chorismate metabolism.metabolika|Superpathway of CMP-sialic acids biosynthesis.metabolika|Superpathway of coenzyme A biosynthesis I.metabolika|Superpathway of coenzyme A biosynthesis II (plants).metabolika|Superpathway of coenzyme A biosynthesis III (mammals).metabolika|Superpathway of cytosolic glycolysis (plants), pyruvate dehydrogenase and TCA cycle.metabolika|Superpathway of D-glucarate and D-galactarate degradation.metabolika|Superpathway of D-myo-inositol (1,4,5)-trisphosphate metabolism.metabolika|Superpathway of demethylmenaquinol-6 biosynthesis I.metabolika|Superpathway of demethylmenaquinol-6 biosynthesis II.metabolika|Superpathway of demethylmenaquinol-8 biosynthesis.metabolika|Superpathway of demethylmenaquinol-9 biosynthesis.metabolika|Superpathway of dimethylsulfone degradation.metabolika|Superpathway of dimethylsulfoniopropanoate degradation.metabolika|Superpathway of diterpene resin acids biosynthesis.metabolika|Superpathway of dTDP-glucose-derived antibiotic building blocks biosynthesis.metabolika|Superpathway of dTDP-glucose-derived O-antigen building blocks biosynthesis.metabolika|Superpathway of ergosterol biosynthesis I.metabolika|Superpathway of ergosterol biosynthesis II.metabolika|Superpathway of ergotamine biosynthesis.metabolika|Superpathway of erythromycin biosynthesis (without sugar biosynthesis).metabolika|Superpathway of erythromycin biosynthesis.metabolika|Superpathway of fatty acid biosynthesis I (E. coli).metabolika|Superpathway of fatty acid biosynthesis II (plant).metabolika|Superpathway of fatty acid biosynthesis initiation (E. coli).metabolika|Superpathway of fatty acids biosynthesis (E. coli).metabolika|Superpathway of fermentation (Chlamydomonas reinhardtii).metabolika|Superpathway of flavones and derivatives biosynthesis .metabolika|Superpathway of formononetin derivative biosynthesis.metabolika|Superpathway of fucose and rhamnose degradation.metabolika|Superpathway of fumitremorgin biosynthesis.metabolika|Superpathway of GDP-mannose-derived O-antigen building blocks biosynthesis.metabolika|Superpathway of geranylgeranyl diphosphate biosynthesis II (via MEP).metabolika|Superpathway of geranylgeranyldiphosphate biosynthesis I (via mevalonate).metabolika|Superpathway of gibberellin biosynthesis.metabolika|Superpathway of gibberellin GA12 biosynthesis.metabolika|Superpathway of glucose and xylose degradation.metabolika|Superpathway of glycerol degradation to 1,3-propanediol.metabolika|Superpathway of glycol metabolism and degradation.metabolika|Superpathway of glycolysis and Entner-Doudoroff.metabolika|Superpathway of glycolysis, pyruvate dehydrogenase, TCA, and glyoxylate bypass.metabolika|Superpathway of glyoxylate bypass and TCA.metabolika|Superpathway of glyoxylate cycle and fatty acid degradation.metabolika|Superpathway of guanine and guanosine salvage.metabolika|Superpathway of guanosine nucleotides degradation (plants).metabolika|Superpathway of guanosine nucleotides de novo biosynthesis I.metabolika|Superpathway of guanosine nucleotides de novo biosynthesis II.metabolika|Superpathway of heme biosynthesis from glutamate.metabolika|Superpathway of heme biosynthesis from glycine.metabolika|Superpathway of heme biosynthesis from

uroporphyrinogen-III.metabolika|Superpathway of hexitol degradation (bacteria).metabolika|Superpathway of hexuronide and hexuronate degradation.metabolika|Superpathway of histidine, purine, and pyrimidine biosynthesis.metabolika|Superpathway of hydrogen production.metabolika|Superpathway of hydrolyzable tannin biosynthesis.metabolika|Superpathway of hyoscyamine and scopolamine biosynthesis.metabolika|Superpathway of indole-3-acetate conjugate biosynthesis.metabolika|Superpathway of inositol phosphate compounds.metabolika|Superpathway of isoflavonoids (via naringenin).metabolika|Superpathway of jasmonoyl-amino acid conjugates biosynthesis.metabolika|Superpathway of L-alanine biosynthesis.metabolika|Superpathway of L-arginine and L-ornithine degradation.metabolika|Superpathway of L-arginine, putrescine, and 4-aminobutanoate degradation.metabolika|Superpathway of L-asparagine biosynthesis.metabolika|Superpathway of L-aspartate and L-asparagine biosynthesis.metabolika|Superpathway of L-citrulline metabolism.metabolika|Superpathway of L-cysteine biosynthesis (mammalian).metabolika|Superpathway of L-isoleucine biosynthesis I.metabolika|Superpathway of L-lysine degradation.metabolika|Superpathway of L-lysine, L-threonine and L-methionine biosynthesis I.metabolika|Superpathway of L-lysine, L-threonine and L-methionine biosynthesis II.metabolika|Superpathway of L-methionine biosynthesis (by sulfhydrylation).metabolika|Superpathway of L-methionine biosynthesis (transsulfuration).metabolika|Superpathway of L-methionine salvage and degradation.metabolika|Superpathway of L-phenylalanine and L-tyrosine biosynthesis.metabolika|Superpathway of L-phenylalanine biosynthesis.metabolika|Superpathway of L-serine and glycine biosynthesis I.metabolika|Superpathway of L-threonine biosynthesis.metabolika|Superpathway of L-threonine metabolism.metabolika|Superpathway of L-tryptophan biosynthesis.metabolika|Superpathway of L-tyrosine biosynthesis.metabolika|Superpathway of linalool biosynthesis.metabolika|Superpathway of linamarin and lotaustralin biosynthesis.metabolika|Superpathway of lipopolysaccharide biosynthesis.metabolika|Superpathway of lipoxygenase.metabolika|Superpathway of megalomicin A biosynthesis.metabolika|Superpathway of melatonin degradation.metabolika|Superpathway of menaquinol-10 biosynthesis.metabolika|Superpathway of menaquinol-11 biosynthesis.metabolika|Superpathway of menaquinol-12 biosynthesis.metabolika|Superpathway of menaquinol-13 biosynthesis.metabolika|Superpathway of menaquinol-6 biosynthesis I.metabolika|Superpathway of menaquinol-7 biosynthesis.metabolika|Superpathway of menaquinol-8 biosynthesis I.metabolika|Superpathway of menaquinol-8 biosynthesis II.metabolika|Superpathway of menaquinol-9 biosynthesis.metabolika|Superpathway of methanogenesis.metabolika|Superpathway of methylglyoxal degradation.metabolika|Superpathway of microbial D-galacturonate and D-glucuronate degradation.metabolika|Superpathway of mycolyl-arabinogalactan-peptidoglycan complex biosynthesis.metabolika|Superpathway of NAD biosynthesis in eukaryotes.metabolika|Superpathway of neomycin biosynthesis.metabolika|Superpathway of nicotinate degradation.metabolika|Superpathway of nicotine biosynthesis.metabolika|Superpathway of oleoresin turpentine biosynthesis.metabolika|Superpathway of ornithine degradation.metabolika|Superpathway of penicillin, cephalosporin and cephamycin biosynthesis.metabolika|Superpathway of pentose and pentitol degradation.metabolika|Superpathway of phenylethylamine degradation.metabolika|Superpathway of phosphatidylcholine biosynthesis.metabolika|Superpathway of phospholipid biosynthesis I (bacteria).metabolika|Superpathway of phospholipid biosynthesis II (plants).metabolika|Superpathway of photosynthetic hydrogen production.metabolika|Superpathway of phylloquinol biosynthesis.metabolika|Superpathway of plastoquinol biosynthesis.metabolika|Superpathway of polyamine biosynthesis I.metabolika|Superpathway of polyamine biosynthesis II.metabolika|Superpathway of polyamine biosynthesis III.metabolika|Superpathway of pterocarpan biosynthesis (via daidzein).metabolika|Superpathway of pterocarpan biosynthesis (via formononetin).metabolika|Superpathway of purine

deoxyribonucleosides degradation.metabolika|Superpathway of purine nucleotide salvage.metabolika|Superpathway of purine nucleotides de novo biosynthesis I.metabolika|Superpathway of purine nucleotides de novo biosynthesis II.metabolika|Superpathway of purines degradation in plants.metabolika|Superpathway of pyridoxal 5'-phosphate biosynthesis and salvage.metabolika|Superpathway of pyrimidine deoxyribonucleoside salvage.metabolika|Superpathway of pyrimidine deoxyribonucleosides degradation.metabolika|Superpathway of pyrimidine deoxyribonucleotides de novo biosynthesis (E. coli).metabolika|Superpathway of pyrimidine deoxyribonucleotides de novo biosynthesis.metabolika|Superpathway of pyrimidine nucleobases salvage.metabolika|Superpathway of pyrimidine ribonucleosides degradation.metabolika|Superpathway of pyrimidine ribonucleosides salvage.metabolika|Superpathway of pyrimidine ribonucleotides de novo biosynthesis.metabolika|Superpathway of quinolone and alkylquinolone biosynthesis.metabolika|Superpathway of rifamycin B biosynthesis.metabolika|Superpathway of roquefortine, meleagrin and neoxaline biosynthesis.metabolika|Superpathway of rosmarinic acid biosynthesis.metabolika|Superpathway of salicylate degradation.metabolika|Superpathway of scopolin and esculin biosynthesis.metabolika|Superpathway of seleno-compound metabolism.metabolika|Superpathway of ss-D-glucuronide and D-glucuronate degradation.metabolika|Superpathway of stearidonate biosynthesis (cyanobacteria).metabolika|Superpathway of steroid hormone biosynthesis.metabolika|Superpathway of sulfate assimilation and cysteine biosynthesis.metabolika|Superpathway of sulfide oxidation (Acidithiobacillus ferrooxidans).metabolika|Superpathway of sulfide oxidation (phototrophic sulfur bacteria).metabolika|Superpathway of sulfide oxidation (Starkeya novella).metabolika|Superpathway of sulfolactate degradation.metabolika|Superpathway of sulfur amino acid biosynthesis (Saccharomyces cerevisiae).metabolika|Superpathway of sulfur metabolism (Desulfocapsa sulfoexigens).metabolika|Superpathway of sulfur oxidation (Acidianus ambivalens).metabolika|Superpathway of taurine degradation.metabolika|Superpathway of testosterone and androsterone degradation.metabolika|Superpathway of tetracycline and oxytetracycline biosynthesis.metabolika|Superpathway of tetrahydrofolate biosynthesis and salvage.metabolika|Superpathway of tetrahydrofolate biosynthesis.metabolika|Superpathway of tetrahydroxyxanthone biosynthesis.metabolika|Superpathway of tetrathionate reduction (Salmonella typhimurium).metabolika|Superpathway of the 3-hydroxypropanoate cycle.metabolika|Superpathway of thiamine diphosphate biosynthesis I.metabolika|Superpathway of thiamine diphosphate biosynthesis II.metabolika|Superpathway of thiamine diphosphate biosynthesis III (eukaryotes).metabolika|Superpathway of thiosulfate metabolism (Desulfovibrio sulfodismutans).metabolika|Superpathway of trichothecene biosynthesis.metabolika|Superpathway of trimethylamine degradation.metabolika|Superpathway of ubiquinol-6 biosynthesis (eukaryotic).metabolika|Superpathway of ubiquinol-8 biosynthesis (prokaryotic).metabolika|Superpathway of UDP-glucose-derived O-antigen building blocks biosynthesis.metabolika|Superpathway of UDP-N-acetylglucosamine-derived O-antigen building blocks biosynthesis.metabolika|Superpathway of unsaturated fatty acids biosynthesis (E. coli).metabolika|Superpathway of vanillin and vanillate degradation.metabolika|Superpathway of Clostridium acetobutylicum acidogenic and solventogenic fermentation.metabolika|Superpathway of Clostridium acetobutylicum acidogenic fermentation.metabolika|Superpathway of Clostridium acetobutylicum solventogenic fermentation.metabolika|Superpathway of N-acetylglucosamine, N-acetylmannosamine and N-acetylneuraminate degradation.metabolika|Superpathway of N-acetylneuraminate degradation.metabolika|Superpathway of S-adenosyl-L-methionine biosynthesis.metabolika|Superpathway polymethylated quercetinquercetagetin glucoside biosynthesis (Chrysosplenium).metabolika|Superpathways of coenzyme A biosynthesis

I.metabolika|Superpathways of coenzyme A biosynthesis III (mammals).metabolika|Syringate degradation.metabolika|Taxadiene biosynthesis (engineered).metabolika|Thiamine salvage II.metabolika|Toluene degradation I (aerobic) (via o-cresol).metabolika|Toluene degradation II (aerobic) (via 4-methylcatechol).metabolika|Toluene degradation III (aerobic) (via p-cresol).metabolika|Toluene degradation IV (aerobic) (via catechol).metabolika|Toluene degradation V (aerobic) (via toluene-cis-diol).metabolika|Toluene degradation VI (anaerobic).metabolika|Trans-lycopene biosynthesis I (bacteria).metabolika|UDP-D-xylose biosynthesis.metabolika|UDP-galactofuranose biosynthesis.metabolika|UDP-sugars interconversion.metabolika|Ureide biosynthesis.metabolika|Vibriobactin biosynthesis.metabolika|Wybutosine biosynthesis.metabolika
- Search Mode: By Formula or Mass

2. By Mass Search Settings:
- Mass Tolerance: 7 ppm

3. By Formula Search Settings:
- Max. # of Predicted Compositions to be searched per Compound: 10

4. Display Settings:
- Max. # Pathways in 'Pathways' column: 30

Processing node 41: Apply Spectral Distance
----
1. Pattern Matching:
- Mass Tolerance: 7 ppm
- Intensity Tolerance [%]: 30
- Intensity Threshold [%]: 0.1
- S/N Threshold: 3
- Use Dynamic Recalibration: True

Processing node 54: Apply mzLogic
----
1. Search Settings:
- FT Fragment Mass Tolerance: 7 ppm
- IT Fragment Mass Tolerance: 0.4 Da
- Max. # Compounds: 0
- Max. # mzCloud Similarity Results to consider per Compound: 10
- Match Factor Threshold: 30

Processing node 40: Search Mass Lists
----
1. Search Settings:
- Mass Lists: Arita Lab 6549 Flavonoid Structure Database.masslist|EFS HRAM Compound Database.masslist|Endogenous Metabolites database 4400 compounds.masslist|LipidMaps Structure Database 2021-09-13.massList
- Mass Tolerance: 7 ppm
- Use Retention Time: False
- RT Tolerance [min]: 2

Processing node 48: Search ChemSpider
----
1. Search Settings:
- Database(s):
    BioCyc
    FooDB
    Human Metabolome Database
    KEGG
    LIPID MAPS
    MassBank
    NIST Spectra
    Serum Metabolome Database
- Search Mode: By Formula or Mass
- Mass Tolerance: 7 ppm
- Max. # of results per compound: 100
- Max. # of Predicted Compositions to be searched per Compound: 3
- Result Order (for Max. # of results per compound): Order By Reference Count (DESC)

2. Predicted Composition Annotation:
- Check All Predicted Compositions: True

Processing node 29: Predict Compositions
----
1. Prediction Settings:
- Mass Tolerance: 7 ppm
- Min. Element Counts: C H
- Max. Element Counts: C90 H190 Br3 Cl4 N10 O18 P6 S5
- Min. RDBE: 0
- Max. RDBE: 40
- Min. H/C: 0.1
- Max. H/C: 3.5
- Max. # Candidates: 15
- Max. # Internal Candidates: 500

2. Pattern Matching:
- Intensity Tolerance [%]: 30
- Intensity Threshold [%]: 0.1
- S/N Threshold: 3
- Min. Spectral Fit [%]: 30
- Min. Pattern Cov. [%]: 90
- Use Dynamic Recalibration: True

3. Fragments Matching:
- Use Fragments Matching: True
- Mass Tolerance: 15 ppm
- S/N Threshold: 3

Processing node 17: Differential Analysis
----
1. General Settings:

- Log10 Transform Values: True

2. Peak Rating Contributions:
- Update Peak Rating: True
- Area Contribution: 3
- CV Contribution: 10
- FWHM to Base Contribution: 5
- Jaggedness Contribution: 5
- Modality Contribution: 5
- Zig-Zag Index Contribution: 5

Processing node 43: Descriptive Statistics

No parameters
